# Engineering synucleinopathy‐resistant human dopaminergic neurons by CRISPR‐mediated deletion of the *SNCA* gene

**DOI:** 10.1111/ejn.14286

**Published:** 2018-12-21

**Authors:** Yixi Chen, Karamjit Singh Dolt, Marco Kriek, Terry Baker, Patrick Downey, Nicola J. Drummond, Maurice A. Canham, Ammar Natalwala, Susan Rosser, Tilo Kunath

**Affiliations:** ^1^ MRC Centre for Regenerative Medicine Institute for Stem Cell Research School of Biological Sciences The University of Edinburgh Edinburgh UK; ^2^ UK Centre for Mammalian Synthetic Biology The University of Edinburgh Edinburgh UK; ^3^ UCB Pharma Ltd. Slough UK; ^4^ UCB BioPharma Sprl. Braine‐l'Alleud Belgium

**Keywords:** CRISPR/Cas9n, disease‐resistant, dopaminergic neurons, hESCs, synucleinopathy

## Abstract

An emerging treatment for Parkinson's disease (PD) is cell replacement therapy. Authentic midbrain dopaminergic (mDA) neuronal precursors can be differentiated from human embryonic stem cells (hESCs) and human induced pluripotent stem cells (iPSCs). These laboratory‐generated mDA cells have been demonstrated to mature into functional dopaminergic neurons upon transplantation into preclinical models of PD. However, clinical trials with human fetal mesenchephalic cells have shown that cell replacement grafts in PD are susceptible to Lewy body formation suggesting host‐to‐graft transfer of α‐synuclein pathology. Here, we have used CRISPR/Cas9n technology to delete the endogenous *SNCA* gene, encoding for α‐synuclein, in a clinical‐grade hESC line to generate *SNCA* ^+/−^ and *SNCA* ^−/−^ cell lines. These hESC lines were first differentiated into mDA neurons, and then challenged with recombinant α‐synuclein preformed fibrils (PFFs) to seed the formation for Lewy‐like pathology as measured by phosphorylation of serine‐129 of α‐synuclein (pS129‐αSyn). Wild‐type neurons were fully susceptible to the formation of protein aggregates positive for pS129‐αSyn, while *SNCA* ^+/−^ and *SNCA* ^−/−^ neurons exhibited significant resistance to the formation of this pathological mark. This work demonstrates that reducing or completely removing *SNCA* alleles by CRISPR/Cas9n‐mediated gene editing confers a measure of resistance to Lewy pathology.

**Abbreviations**
6‐OHDA: 6‐hydroxydopamine
AA: ascorbic acid
AST18: alpha‐synuclein triplication 18 iPSC line
BDNF: brain‐derived neurotrophic factor
Cas9n: Cas9‐nickase (D10A)
CRISPR: clustered regularly interspaced short palindromic repeat
FGF8b: fibroblast growth factor 8b
FTIR: Fourier‐transform infrared
GBA: glucocerebrosidase
GDNF: glial cell line‐derived neurotrophic factor
gRNA: guide RNA
HEK 293: human embryonic kidney 293 cell line
hESCs: human embryonic stem cells
HIV: human immunodeficiency virus
HRP: horseradish peroxidase
iPSCs: induced pluripotent stem cells
mDA: midbrain dopaminergic
MPTP: 1‐methyl‐4‐phenyl‐1,2,3,6‐tetrahydropyridine
NDM: neural differentiation medium
NIM: neural induction medium
NPM: neural patterning medium
PD: Parkinson's disease
PFFs: preformed fibrils
pS129‐αSyn: phosphorylated serine‐129‐α‐synuclein
SHH: sonic hedgehog
SNCA: synuclein, alpha gene (human)
Snca, synuclein, alpha gene (mouse); Sncb: synuclein, beta gene (mouse)
Sncg: synuclein, gamma gene (mouse)
T7E1: T7 endonuclease I
TBS‐N: tris‐buffered saline with Nonidet P‐40
TH: tyrosine hydroxylase
VM: ventral mesencephalon

## 1 INTRODUCTION

Parkinson's disease (PD) is a common neurological disorder that is caused by the loss or dysfunction of specific neuronal cell types. The dopaminergic neurons of the *substantia nigra pars compacta* that innervate the dorsal striatum are significantly affected in this condition. The degeneration of the nigrostriatal dopaminergic pathway is largely responsible for the motor symptoms of PD.

An experimental therapy for PD is transplantation of fetal ventral mesencephalic cells into the striatum (Lindvall et al., 1988, 1990, 1994). Although some patients experienced long‐term alleviation of motor symptoms (Li et al., 2016; Ma et al., 2010), a significant proportion suffered from graft‐induced dyskinesias (Hagell et al., 2002; Piccini et al., 2005), which was attributed to serotonergic hyperinnervation from the graft (Politis et al., 2010). Furthermore, most grafts older than 10 years acquired Lewy body pathology suggesting that host‐to‐graft spread of disease may be occurring (Kordower, Chu, Hauser, Freeman, & Olanow, 2008; Li et al., 2008; Mendez et al., 2008). The burden of Lewy bodies in the graft correlated with a decrease in the symptomatic benefit to the patient (Chu & Kordower, 2010). These clinical observations highlight the need for cell therapies that are resistant to the formation of Lewy bodies, which are predominantly composed of the protein α‐synuclein (Spillantini et al., 1997). Such disease‐resistant cells will be particularly important for patients with young‐onset Parkinson's or genetic forms of the condition with substantial α‐synuclein burden, such as *SNCA* multiplications (Farrer et al., 2004) and *GBA* mutation carriers (Neumann et al., 2009).

In cell culture and animal models of PD, synucleinopathy can be initiated by inoculation with preformed fibrils (PFFs) of recombinant α‐synuclein protein. In primary rodent neurons, molecular pathology is observed by monitoring the phosphorylation of α‐synuclein at position serine‐129. This is a prominent posttranslational event that is found in Lewy bodies, and is an early event during the aggregation of α‐synuclein (Fujiwara et al., 2002). If neurons lack endogenous expression of α‐synuclein they are not susceptible to α‐synuclein PFF seeding (Volpicelli‐Daley et al., 2011), and it has been described that neurons with low endogenous expression of α‐synuclein are spared in PD (Braak, Ghebremedhin, Rüb, Bratzke, & Del Tredici, 2004). Furthermore, when *Snca* ^−/−^ mice were stereotactically injected with α‐synuclein PFFs they exhibited no signs of synucleinopathy or neurodegeneration unlike their wild‐type counterparts (Luk et al., 2012).

The differentiation of human embryonic stem cells (hESCs) and induced pluripotent stem cells (iPSCs) into midbrain dopaminergic (mDA) neurons is now well understood and described (Kirkeby et al., 2017; Kriks et al., 2011), and the route to clinical trials for hESC/iPSC‐derived mDA cell therapy for Parkinson's has been mapped out (Barker, Parmar, Studer, & Takahashi, 2017). However, these grafts will express wild‐type levels of α‐synuclein protein making them susceptible to synucleinopathy with a similar kinetics to fetal mesencephalic grafts. Here, we show that deleting alleles of the *SNCA* gene in hESCs and iPSCs reduces or eliminates α‐synuclein expression from mDA neurons and confers resistance to α‐synuclein PFF induced formation of Lewy‐like pathology. This work sets out a strategy to produce disease‐resistant mDA grafts for Parkinson's that will last the life time of the individual, and will be particularly important for patients with an aggressive synucleinopathy.

## 2 MATERIALS AND METHODS

### 2.1 hESC and iPSC culture

Approval for the use of hESCs used in this study was granted by the MRC Steering Committee for the UK Stem Cell Bank and for the Use of Stem Cell Lines (ref, SCSC13‐19). RC17 hESCs (~passages 25‐30) and AST18 iPSCs were maintained in self‐renewing conditions on Laminin‐521 (L521, 5 μg/ml, Biolamina) coated 6‐well plates (Corning), and fed daily with StemMACS iPS‐Brew XF (iPS‐B, Miltenyi Biotec). Every 2 or 3 days, once the hESCs reached 70%–90% confluency, the cells were passaged as clumps with EDTA (0.5 mM, Thermo Fisher Scientific) at a split ratio of 1:3 to 1:8. See Supporting Information Table S1 for all catalogue numbers.

### 2.2 Cloning of gRNA plasmids

For each gRNA, a 100 bp double‐stranded DNA fragment was made by PCR of a pair of partially complementary single‐stranded DNA, of which the complementary sequence was the gRNA (Mali et al., 2013). The fragment was then cloned into *Bbs*I‐HF (NEB) digested pSpCas9n‐2A‐Puro plasmid (PX462, Addgene) (Ran et al., 2013) by Gibson assembly and transfected into Stbl3 competent *E. coli* (Thermo Fisher Scientific). The PX462 plasmid was obtained under a standard Addgene MTA (Order 152516) with the Provider Organization, Broad Institute, Inc.

CRISPR gRNA sequences:

5G1: tgaattcctttacaccacac
3G1: gccatggatgtattcatgaa
5G4: acaagcaccaaactgacatt
3G4: ataatcaatactctaaatgc

### 2.3 *SNCA* knockout with CRISPR/Cas9n

RC17 hESCs or AST18 iPSCs (8 × 10^5^ cells) were transfected with 1 μg of each of the 4 gRNA plasmids and GFPiPuroR (a GFP indicator plasmid) using Amaxa^™^ Human Stem Cell Nucleofector^™^ Kit 1 (Lonza) and programme B‐016 on Nucleofector^®^ II (Lonza). 24 hr after transfection, puromycin (0.5 μg/ml, Sigma) selection (16 hr) was applied for cells on Matrigel (Thermo Fisher Scientific) or 1.0 μg/ml puromycin for cells on L521 (5 μg/ml). Clones were picked 8–14 days after transfection and expanded on L521‐coated 96‐well plates.

### 2.4 PCR genotyping

DNA was extracted from hESC clones using 1 hr incubation at 98°C in Solution I (25 mM NaOH and 0.2 mM EDTA) followed by termination with Solution II (40 mM Tris‐HCl). Three PCR reactions flanking the targeted region were performed using Q5^®^ High‐Fidelity DNA Polymerase (NEB) with primers to produce products of 449 bp, 943 bp, and 1,927 bp.

PCR primer sequences (5′–3′):

449 bp Forward: cacactttggagggtttctc

449 bp Reverse: ctggaaaagcaaacagtcgc

943 bp Forward: cagcttccatgcttctaactc

943 bp Reverse: ctttatacacatcacaggggc

1,927 bp Forward: ccacaagggctgagagat

1,927 bp Reverse: tggtcatcctccacctga

PCR products were electrophoresed in 2% agarose (Thermo Fisher Scientific) gels with Quick‐Load^®^ 100 bp DNA ladder (NEB) or 1 kb DNA ladder (NEB).

### 2.5 Western blotting

Cells were lysed with RIPA Lysis Buffer System (Santa Cruz) according to manufacturer's instructions. Following quantification of protein concentration using the BCA assay (Thermo Fisher Scientific), 15 μg of protein was incubated with NuPAGE^™^ LDS loading dye (1:4, Thermo Fisher Scientific) and NuPAGE^™^ Sample Reducing Agent (1:10, Thermo Fisher Scientific) at 70°C for 10 min and then loaded onto NuPAGE^™^ 4%–12% Bis‐Tris Protein Gel (Thermo Fisher Scientific) with SeeBlue^™^ Plus2 ladder (Thermo Fisher Scientific). The protein was electrophoresed and transferred onto Amersham^™^ Protran^™^ Premium 0.45 μm NC nitrocellulose membrane (Amersham). The membrane was fixed with 0.4% paraformaldehyde (PFA, Fisher) for 30 min and blocked with 10% blocking‐grade blocker (BioRad) in 0.05% TBS‐N. Mouse anti‐α‐synuclein primary antibody (BD, 1:1,000) was added in 5% blocking‐grade blocker in 0.05% TBS‐N and rocked at 4°C overnight. Following secondary antibody (HRP conjugated anti‐mouse IgG, Promega) incubation, Pierce^™^ ECL Western Blotting Substrate (Thermo Fisher Scientific) was applied before image acquisition. After antibodies were removed with Restore^™^ PLUS Western Blot Stripping Buffer (Thermo Fisher Scientific), the membrane was blocked and reblotted with anti‐β‐actin antibody (HRP conjugate, Abcam) in 5% blocking‐grade blocker in 0.05% TBS‐N. Pierce^™^ ECL Western Blotting Substrate was applied prior to image acquisition.

### 2.6 TOPO‐cloning and DNA sequencing

PCR products of putative *SNCA* knockout clones were purified with Wizard^®^ SV Gel and PCR Clean‐Up System (Promega), incubated with Taq DNA polymerase at 72°C for 10 min, and inserted into pCR^™^4‐TOPO^®^ vector (TOPO^®^ TA Cloning^®^ Kit for Sequencing, Thermo Fisher Scientific) according to manufacturer's instructions. The plasmids were respectively transfected into DH5α competent *E. coli* (NEB) according to manufacturer's instructions. Carbenicillin (Fisher) was used at 100 μg/ml in LB/agar plates for selection of transformed cells. The next day, clones were picked into liquid LB‐containing 100 μg/ml carbenicillin. After overnight incubation, plasmids were isolated with QIAprep Spin Miniprep Kit (QIAGEN). The purified plasmids were digested with *Eco*RI (NEB) and electrophoresed on 1% agarose gel. The plasmids of potential *SNCA* knockout clones (using T7 primer) and the purified PCR product of the potential α‐synuclein homozygous knockout clones were sent for Sanger sequencing at the MRC PPU DNA Sequencing and Services (Dundee).

### 2.7 Off‐target analysis

The top two potential off‐target sites for 5G1 and 3G1 gRNAs were identified using the CRISPR design webtool (http://crispr.mit.edu/).

Potential off‐target sites:

5G1 off‐target #1: tgtattccagtacaccacac (chr15:+46664956)
5G1 off‐target #2: tgagttcctctccaccacac (chr10:+80560295)
3G1 off‐target #1: gccatggctgtattcaagaa (chr9:+93313553)
3G1 off‐target #2: gccctgggtgcattcatgaa (ch22:+25774724)
PCR primer sequences and product sizes for off‐target analysis (5′–3′):
5G1 OffTgt1F: ccagacttcacaaccaccca, R: caggatcaagtggggtgtgg (370 bp)
5G1 OffTgt2F: tggcagcaggtttcctactc, R: gctcttggttgtactggcct (527 bp)
3G1 OffTgt1F: gcttgggcaacacaatgaga, R: acctgtctatccgtctgccc(532 bp)
3G1 OffTgt2F: catgctctgcctcaaccaga, R: cagagaggcaggcatgtgaa (311 bp)

PCR products were generated and subjected to a T7E1 assay to determine if any of the potential off‐target sites sustained a deletion or insert. Briefly, PCR products were dissociated and re‐annealed before addition of T7E1 (10U, NEB) in NEBuffer^™^ 2 (NEB) and incubated at 37°C for 20 min. Reaction products were electrophoresed in 2% agarose (Thermo Fisher Scientific) gels with Quick‐Load^®^ 100 bp DNA ladder (NEB).

### 2.8 Midbrain dopaminergic neuronal differentiation

For differentiation, hESCs were seeded at a density of 40,000 cells/cm^2^ onto Laminin‐111 (L111, 5 μg/ml, Biolamina) coated plates according to an adapted published protocol (Kirkeby et al., 2017). Cells were differentiated from day 0 to day 4 in neural induction medium (NIM) which was made up of 50% DMEM/F12 (Thermo Fisher Scientific) + 50% Neurobasal Media (Thermo Fisher Scientific) + B27 supplement (without Vitamin A, 1:50, Thermo Fisher Scientific) + N2 supplement (1:100, Thermo Fisher Scientific) + l‐Glutamine (2 mM, Thermo Fisher Scientific). Y27632 (Y2, 10 μM, Tocris) was present in medium from day 0 to day 2. From day 0 to day 9, SB431542 (SB, 10 μM, Millipore), LDN‐193189 (LDN, 100 ng/ml, Miltenyi Biotec), Shh‐C24II (SHH, 600 ng/ml, R&D Systems) and CHIR99021 (CHIR, 1.3 μM, Miltenyi Biotec) were added. From day 4 to day 11, medium was changed to neural patterning medium (NPM) which consisted of 50% DMEM/F12 + 50% Neurobasal Media + B27 supplement (1:100) + N2 supplement (1:200) + l‐Glutamine (2 mM). From day 9 to day 16, FGF8b (100 ng/ml, R&D Systems) and heparin (1 μg/ml, Sigma) were added to the medium. On day 11, cells were lifted with Accutase (Sigma) and replated to L111‐coated 48‐well plates at a density of 800,000 cells/cm^2^. From day 11, cells were fed with neural differentiation medium (NDM) which was Neurobasal Media + B27 supplement (1:50) + l‐Glutamine (2 mM) and supplemented with ascorbic acid (AA, 0.2 mM, Sigma), brain‐derived neurotrophic factor (BDNF, 20 ng/ml, Peprotech) and glial cell line‐derived neurotrophic factor (GDNF, 10 ng/ml, Peprotech), as well as FGF8b and heparin. On day 16, cells were lifted with Accutase and replated in L111‐coated 48‐well plates at a density of 800,000 cells/cm^2^. From day 16, cells were cultured in NDM supplemented with dibutyryl cyclic AMP (dcAMP, 0.5 mM, Sigma) and DAPT (1 μM, Tocris) in addition to BDNF, GDNF, and AA. Around day 25, cells were lifted with Accutase and re‐plated onto poly‐ornithine (0.0015%, Sigma) + L111‐coated 8‐well glass bottom plates (ibidi) at 25,000 cells/cm^2^. Cells were fed on days 2, 4, 7, 9, 11, 14, 16, every 2–3 days after day 16, and weekly after re‐plated onto glass bottom plates.

### 2.9 Gene expression analysis by RT‐qPCR

Total RNA was isolated and DNaseI‐treated from self‐renewing hESCs and day 44 mDA neurons with the MasterPure^™^ Complete DNA and RNA Purification Kit (Epicentre) following the manufacturer's instructions. cDNA was synthesized from 500 ng of total RNA using the SuperScript^™^ IV Reverse Transcriptase (Thermo Fisher Scientific). qPCR was performed using the Roche LightCycler^®^ 480 System with the Universal Probe Library (UPL) (Roche). The Roche UPL Assay design centre was used to design intron‐spanning primers with a specific UPL probe for each gene (*TBP* F‐gaacatcatggatcagaacaaca R‐atagggattccgggagtcat Probe 87; *NANOG* F‐tctccaacatcctgaacctca R‐ttgctattcttcggccagtt Probe 87; *OCT4* F‐tgccgtgaaactggagaag, R‐gcttggcaaattgttcgagt Probe 78; *DAT* F‐ agactgcccgaagtgtgc, R‐gcagtttcccgttacaccaa Probe 14; *NURR1* F‐atttcctcgaaaacgcctgt, R‐catactgcgcctgaacacaa Probe 41; *SOX6* F‐gcttctggactcagcccttta, R‐ggccctttagcctttggtta Probe 50; *VMAT2* F‐cgggattctgcatcatgttt, R‐tggcaatcagcaggaagg Probe 67). Reactions (10 μl) containing cDNA, primers, UPL Probe, LightCycler^®^ 480 Probes Master mix (Roche) and PCR water were performed in 386‐well plates as described in the manufacturer's instructions. The data were normalized to levels of *TATA‐binding protein* (*TBP*) and the data of technical replicates were plotted.

### 2.10 Immunostaining

Cells were fixed with 4% PFA and blocked with 2% donkey serum (Sigma) in PBS‐T (0.1% Triton X‐100 (Fisher) in PBS (Thermo Fisher Scientific)). Primary antibodies used include tyrosine hydroxylase (1:1,000, rabbit, Millipore), FOXA2 (1:100, goat, Santa Cruz), α‐synuclein (1:250, mouse IgG2a, Abcam), α‐synuclein (1:250, mouse IgG1, BD), phosphoserine‐129 α‐synuclein (pS129‐αSyn, 1:1,000, rabbit, Abcam) and β‐III tubulin (1:1,000, mouse IgG2b, Abcam). Secondary antibodies including donkey anti‐rabbit IgG Alexa Fluor‐488 (Thermo Fisher Scientific), donkey anti‐goat IgG Alexa Fluor‐568 (Thermo Fisher Scientific), donkey anti‐mouse IgG Alexa Fluor‐647 (Abcam), goat anti‐mouse IgG2a Alexa Fluor‐488 (Thermo Fisher Scientific), goat anti‐mouse IgG1 Alexa Fluor‐488 (Thermo Fisher Scientific), goat anti‐mouse IgG2b Alexa Fluor‐647 (Thermo Fisher Scientific) were used at 1:1,000, while DAPI (Thermo Fisher Scientific) was used at 1:10,000. Images were acquired on the Axio Observer (Zeiss) or the Eclipse Ti‐E (Nikon) microscope.

### 2.11 Flow cytometry

For flow cytometry, cells were harvested with Accutase and washed with NDM. Cells were re‐suspended with flow cytometry buffer, which consisted of DPBS (Sigma) + 2% fetal calf serum (Thermo Fisher Scientific), and stained with rat anti‐CORIN antibody (R&D Systems, 1:200). Cells were washed with flow cytometry buffer and stained with donkey anti‐rat IgG Alexa Fluor‐488 (Thermo Fisher Scientific). Flow cytometry was performed on BD FACS Calibur (BD Biosciences) and analysed on the FlowJo software program (FlowJo, LLC).

### 2.12 Production of α‐synuclein preformed fibrils (PFFs)

Human *SNCA* cDNA was cloned into a plasmid for mammalian expression, pMKC451, under the control of a CMV promoter. This plasmid (1 mg) was transfected into Expi293 cells (>97% viability, 2,500 million cells) grown in Expi293 Expression Medium (A1435101, Gibco) using Expi293fectamine (1956760, Gibco) and Opti‐MEM (11058021, Gibco), according to manufacturer's instructions. The culture was incubated at 37°C, 120 rpm, 8% CO_2_ (Kuhner Shaker X) and after 17 hr Enhancers 1 and 2 (1956760, Gibco) were added as specified. The culture was harvested 4 days after transfection (typical viability 40%) by centrifugation (90 min, 5,000 *g*, JS4.2 rotor in J6‐MI Beckman) and filter sterilised through a 0.22 μm filter (Millipak Gammagold, Millipore) using a Sartobran P 0.45 + 0.22 μm prefilter (Sartorius Stedim).

Supernatant was diluted with an equal volume 20 mM Tris (T1503, Sigma)/HCl (20252.335, VWR Chemicals) pH 8.0 made up in FlowFusor pyrogen free water (FBP7205, Fresenius Kabi) and was passed over a 2 × 5 ml HiTrap Q FF column (17‐5156‐01, GE Healthcare). Bound protein was eluted with a gradient to 0.4M NaCl (S/3160/60, Fisher Chemical) in 20 mM Tris/HCl over 20 column volumes. Fractions with α‐synuclein were pooled, concentrated using a Centriprep 10K, 10,000 NMWL spin filter (4305, Millipore) and buffer exchanged into 20 mM Tris/HCl pH8.0 using a HiPrep 26/10 desalting column (GE Healthcare). Anion exchange chromatography was repeated using a MonoQ 10/100GL column (GE Healthcare) and fractions with α‐synuclein were pooled, concentrated and purified over a HiLoad 26/600 Superdex 75 column (GE Healthcare) equilibrated in PBS (P5368, Sigma) made up in FlowFusor pyrogen free water (FBP7205, Fresenius Kabi). Analysis throughout the purifications was performed by 4%–12% NuPage Bis/Tris gel electrophoresis using SeeBlue Plus2 prestained molecular weight marker. The final pool was concentrated, filter sterilized (0.22 μm Millex GV, Millipore) to 5–10 mg/ml protein and stored at −80°C in BioPur Safe‐Lock tubes (0030121.589, Eppendorf).

Protein was analysed by HuPage gel electrophoresis and a single protein species was observed (Supporting Information Figure S5A), and electrospray mass spectrometry (ESI+) gave a deconvoluted mw of 14502 Da (mw calc. 14501 Da for N‐acetylated species). Size exclusion chromatography with multi‐angle static light scattering (SEC‐MALS) (data not shown), JC‐1 fluorescence (Lee et al., 2009), and attenuated total reflectance Fourier‐transform infrared (ATR‐FTIR) spectrometry analysis were used to confirm that the recombinant human α‐synuclein was monomeric (Supporting Information Figure S5B,C). Endotoxin level was measured by Limulus Amebocyte Lysate test (PTS20F, Charles River Laboratories). The protein was not phosphorylated at position serine‐129 as determined by the pS129‐αSyn antibody (Abcam).

The PFFs were prepared by shaking the purified human α‐synuclein monomer (7–10 mg/ml, 0.50 ml) in 1.5‐ml BioPur Eppendorf vials at 1,200 rpm, 37°C for 10 days, using a VorTemp56 incubator (Labnet). The resulting material was analysed using JC‐1 (T3168, Molecular Probes) fluorescence (Lee et al., 2009) on a Varian Cary Eclipse fluorimeter, 100 *g* ultracentrifugation (Beckman Optima TLX with a TLA‐120.1 rotor) sedimentation to measure residual monomer by NuPage gel electrophoresis and ATR‐FTIR spectrometry analysis (Tensor 27, Bruker) (Kaylor et al., 2005). Material with a main JC‐1 fluorescence absorbance at 540 nm (Supporting Information Figure S5B), <5% monomer as judged by NuPage electrophoresis of the ultracentrifugation supernatant and an ATR‐FTIR absorption between 1,625 and 1,630 cm^−1^ (Supporting Information Figure S5C) was sonicated for 4 × 10 seconds at 2.5 mg/ml PBS in 1.5‐ml BioPur Eppendorf vial on ice using a Soniprep sonicator (MSE). The protein solution was aliquoted and frozen on dry ice and stored at −80°C in BioPur Eppendorf vials.

### 2.13 PFF seeding and pS129‐αSyn quantification

On day 44 of mDA differentiation, neurons were exposed to α‐synuclein PFFs or monomers (5 μg/ml) by replacing consumed media with feeding media containing the α‐synuclein protein. Half of the consumed media were replaced weekly until 5 weeks after seeding. After immunostaining with pS129‐αSyn and β‐III tubulin, neurons were imaged with Axio Observer (Zeiss) or Eclipse Ti‐E (Nikon) and deconvoluted with Huygens (Scientific Volume Imaging). Images were processed with Fiji (National Institutes of Health): Z‐project images of the two channels were created after setting brightness and contrast, followed by converting to binary images. In order to only quantify pS129‐αSyn structures within β‐III tubulin‐positive neurons, a Boolean “AND” function of the two binary images was performed on Fiji. The area of pS129‐αSyn immunostaining divided by the total area of β‐III tubulin was used for quantification. Statistical analysis was performed in PrismGraphPad software to compare experimental conditions. A two‐tailed, unpaired nonparametric analysis was performed using the Mann–Whitney *U* test.

## 3 RESULTS

### 3.1 CRISPR‐mediated deletion of *SNCA* in hESCs

**Figure 1:**
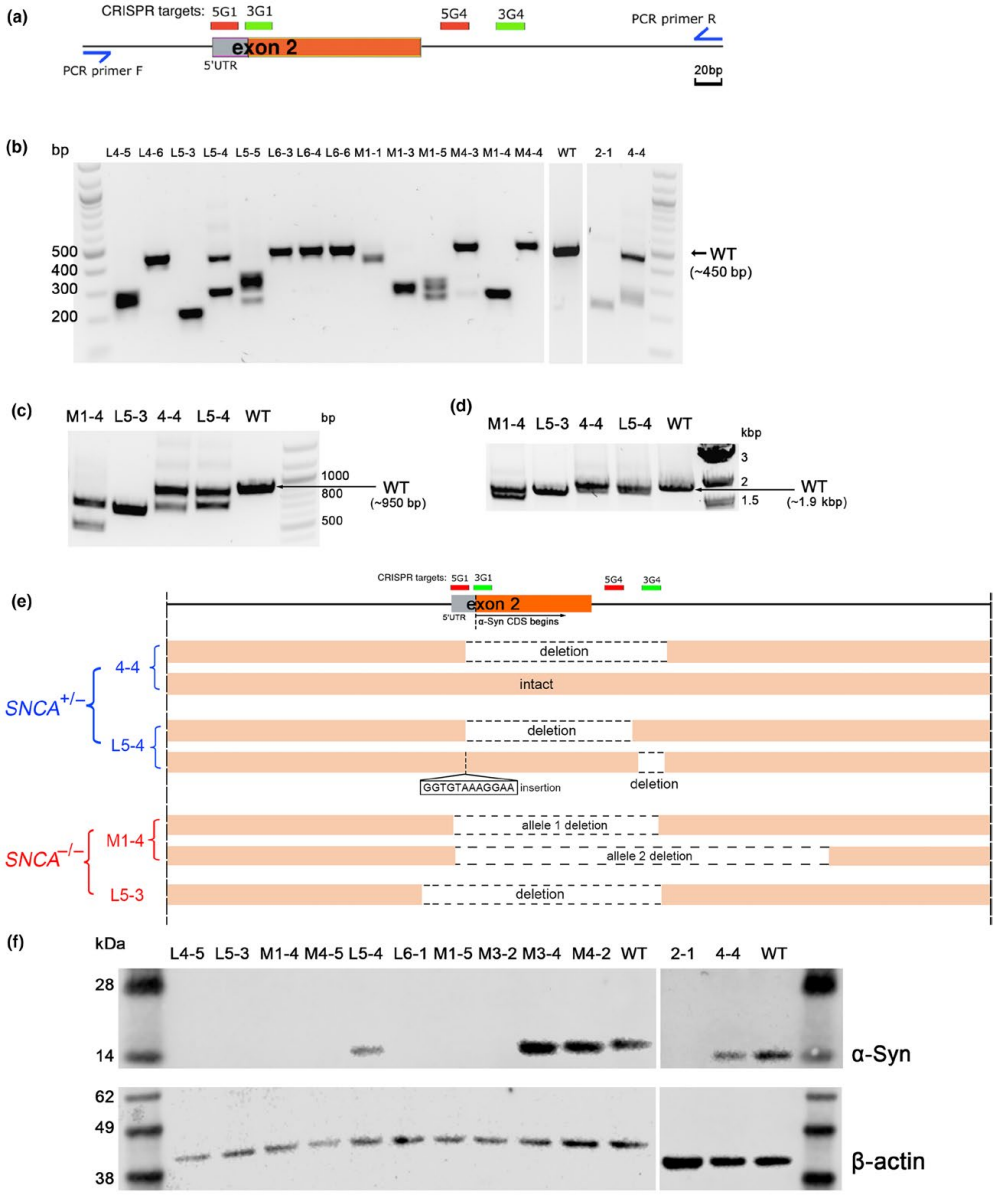
Generation of *SNCA* ^+/−^ and *SNCA* ^−/−^ human embryonic stem cell (hESC) clones and characterization their CRISPR deletions. (a) A schematic view of *SNCA* exon 2 with the location of CRISPR targeting gRNAs and PCR screening primers indicated. (b) PCR screening results of a subset (16) of the clonal hESC lines analysed on 3 separate gels. The expected wild‐type (WT) band size is 449 bp. (c, d) Long‐range PCR of four putative *SNCA* ^+/−^ or *SNCA* ^−/−^ clones for larger regions around the targeted sites: expected WT band sizes are: (c) 943 bp and (d) 1,927 bp. (e) Schematic summary of sequencing results for two *SNCA* ^+/−^ clones (L5‐4 and 4‐4) and two *SNCA* ^−/−^ clones (M1‐4 and L5‐3). (f) Western blotting results for α‐synuclein (α‐Syn) protein for twelve putative *SNCA* ^+/−^ or *SNCA* ^−/−^ clones analysed on two separate gels. Eight α‐Syn‐null clones were confirmed, and two putative *SNCA* ^+/−^ clones (L5‐4 and 4‐4) had a reduced level of α‐Syn expression. β‐actin was blotted as a loading control

A series of guideRNAs (gRNAs) flanking exon 2, the first coding exon, of human *SNCA* were designed using the Zhang Lab CRISPR DESIGN webtool (http://crispr.mit.edu). The oligonucleotides encoding for the top 2 pairs of gRNAs at the 5′ end of *SNCA* exon 2 and the top 2 pairs of gRNAs at the 3′ end of *SNCA* exon 2 were cloned in the pSpCas9n‐2A‐Puro plasmid (Ran et al., 2013) and tested for deletion efficiency in HEK 293 cells (data not shown). We employed the nickase mutant of Cas9, Cas9‐D10A (Cas9n), to avoid off‐target double‐strand breaks in the cells (Ran et al., 2013). Based on cutting efficiency in HEK 293 cells, we decided upon 5G1 and 3G1 gRNAs to target the 5′ end of *SNCA* exon 2, and 5G4 and 3G4 gRNAs to target the 3′ end (Figure 1a). We designed a PCR screening strategy to detect hESC clones with a complete deletion of exon 2. We used RC17 hESCs as the parental line, since it has all the regulatory paperwork in place to be used as a cell therapy in clinical trials (De Sousa et al., 2016). Here, hESCs were electroporated with four gRNA Cas9n plasmids and clones selected in puromycin. After replica plating of the clonal hESC lines, genomic DNA was isolated from 51 clones and analysed for exon 2 deletion by PCR (Figure 1a). Nine putative *SNCA* ^−/−^ clones and four putative *SNCA* ^+/−^ clones were identified by this method. The wild‐type PCR band is expected to be ~450 bp and the knock‐out *SNCA* alleles were typically between 400 bp and 200 bp in size for this initial PCR screening (Figure 1b). A collection of putative knock‐out clones were investigated further with long‐range PCR genotyping strategies to determine if larger deletions could be detected (Figure 1c,d). This revealed that clone M1‐4 harboured two unique knock‐out alleles, only the first of which could be detected by the original PCR genotyping method. Clone L5‐3 appeared to have only a single deletion allele, and no wild‐type alleles (Figure 1c,d). The PCR products of four hESC clones were TOPO‐cloned and sent for Sanger sequencing to determine the precise deletion events (Supporting Information Figures S1–S3). Clones 4‐4 and L5‐4 were putative *SNCA* ^+/−^ cell lines. Clone 4‐4 had one intact allele, and a 210‐bp deletion in the second allele removing most of exon 2 including the ATG start site. Clone L5‐4 harboured a 175‐bp deletion in one allele removing the ATG, and a 12‐bp insertion in the 5′ UTR and 28‐bp deletion in intron 2 of the second allele (Figure 1e, Supporting Information Figure S2). Clones M1‐4 and L5‐3 were putative *SNCA* ^−/−^ cell lines. Clone M1‐4 had a 214‐bp deletion in one allele, and a 428‐bp deletion in the second allele, both of which remove exon 2 and the ATG start codon (Figure 1e, Supporting Information Figure S3). Clone L5‐3 harboured a 250‐bp deletion removing exon 2 and an absence of any wild‐type allele. All three PCR genotyping reactions only detected a single allele in Clone L5‐3 suggesting the deletions are nearly identical on both alleles, or that the second allele has a deletion beyond the detection of the ~1.9 kb PCR reaction (Figure 1d). We investigated the four most likely off‐target cleavage sites in the genome using a T7 endonuclease I (T7E1) assay. We did not observe any off‐target cleavage at these sites as anticipated from using the Cas9n system (Supporting Information Figure S4). We then checked α‐synuclein protein expression in these clones and others by western blotting of undifferentiated hESC lysates. Clones M1‐4, L5‐3, and other clones were confirmed to lack any detectable α‐synuclein expression, while putative *SNCA* ^+/−^ clones, 4‐4 and L5‐4 had reduced expression (Figure 1f). This suggested that the small insertion on the 5′UTR and 28‐bp deletion in intron 2 of Clone L5‐4 did not affect α‐synuclein expression.

### 3.2 Normal midbrain dopaminergic differentiation of *SNCA* targeted hESCs

**Figure 2:**
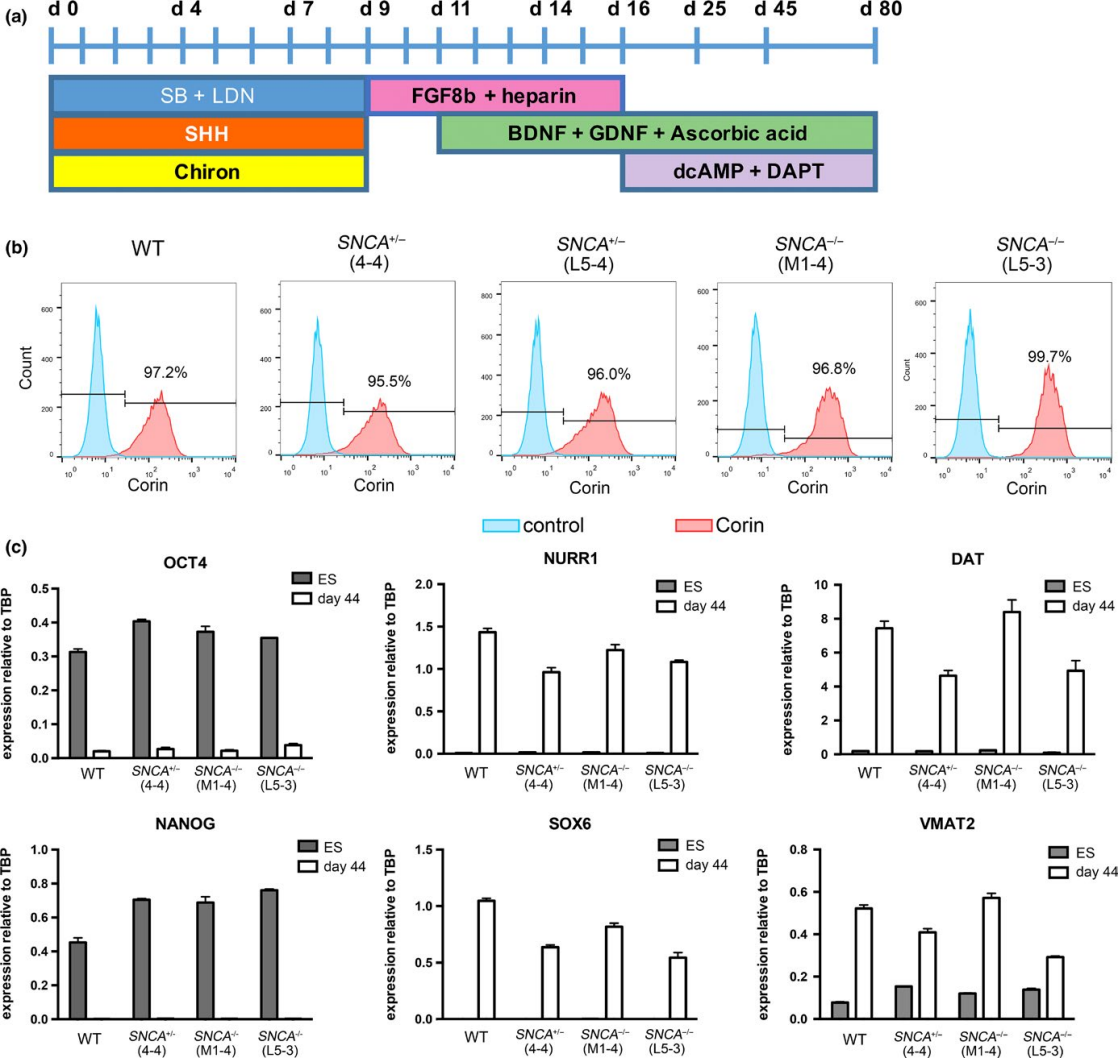
Midbrain dopaminergic (mDA) neuronal differentiation of hESCs is unaffected by deletion of the *SNCA* gene. (a) Floor plate mDA differentiation protocol. (b) Corin flow cytometry of day 16 mDA progenitors differentiated from *SNCA* ^+/−^, *SNCA* ^−/−^ or WT hESCs: Corin (red) and no primary antibody control (blue). (c) RT‐qPCR of hESCs and day 44 mDA neurons for pluripotency markers, *OCT4* and *NANOG*, and dopaminergic markers, *NURR1*,*DAT*,*SOX6*, and *VMAT2*. Gene expression levels were normalized to Tata‐binding protein (*TBP*), plotted as mean ± standard deviation of technical replicates

**Figure 3:**
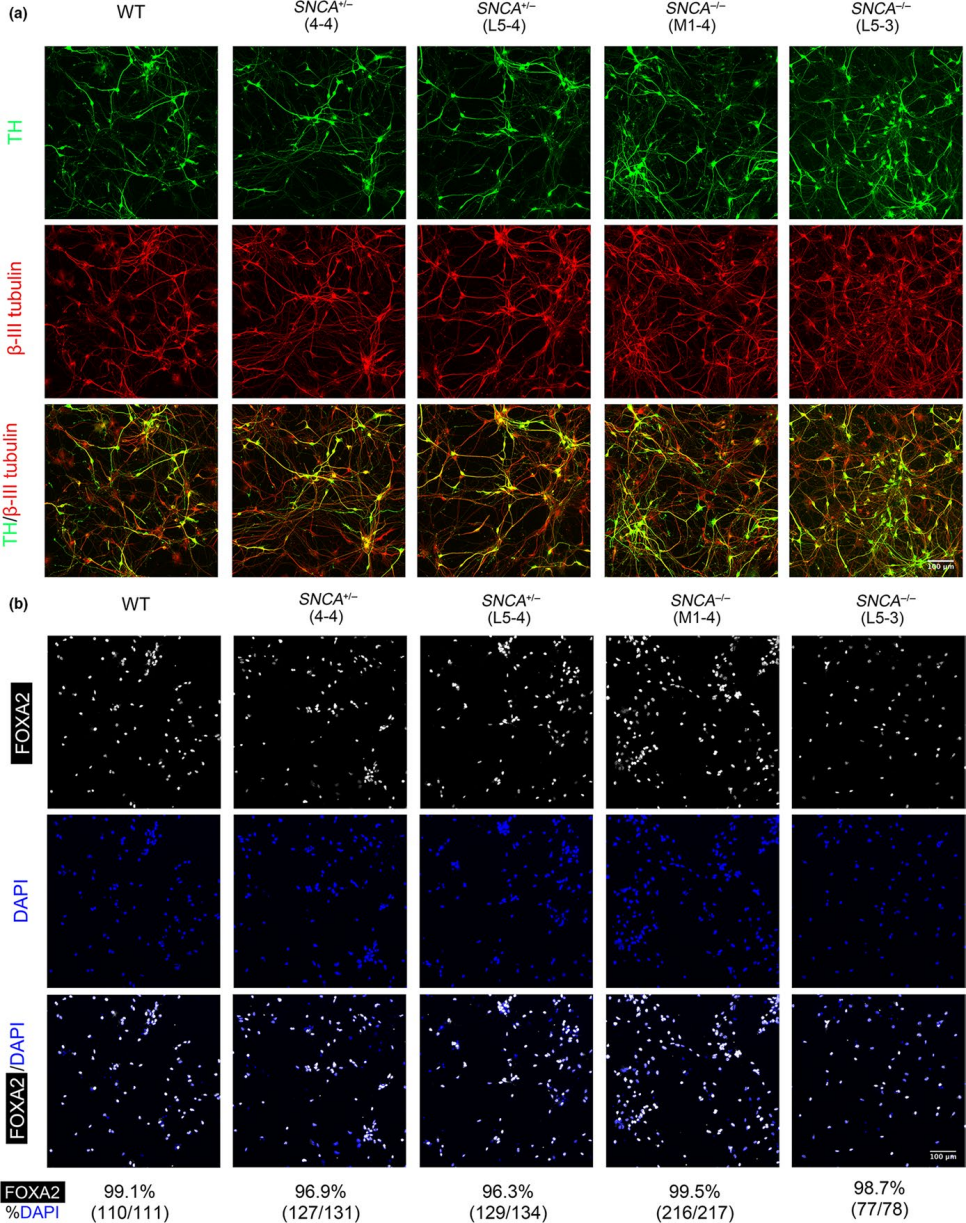
Deletion of *SNCA* does not affect expression of tyrosine hydroxylase (TH) or FOXA2 in mDA neurons. (a) Immunostaining of day 44 mDA neurons differentiated from *SNCA* ^+/−^, *SNCA* ^−/−^ or WT hESCs; TH (green)/β‐III tubulin (red). (b) Day 44 mDA neurons immunostained for FOXA2 (white) and quantified as a percentage of total cells (DAPI, blue)

**Figure 4:**
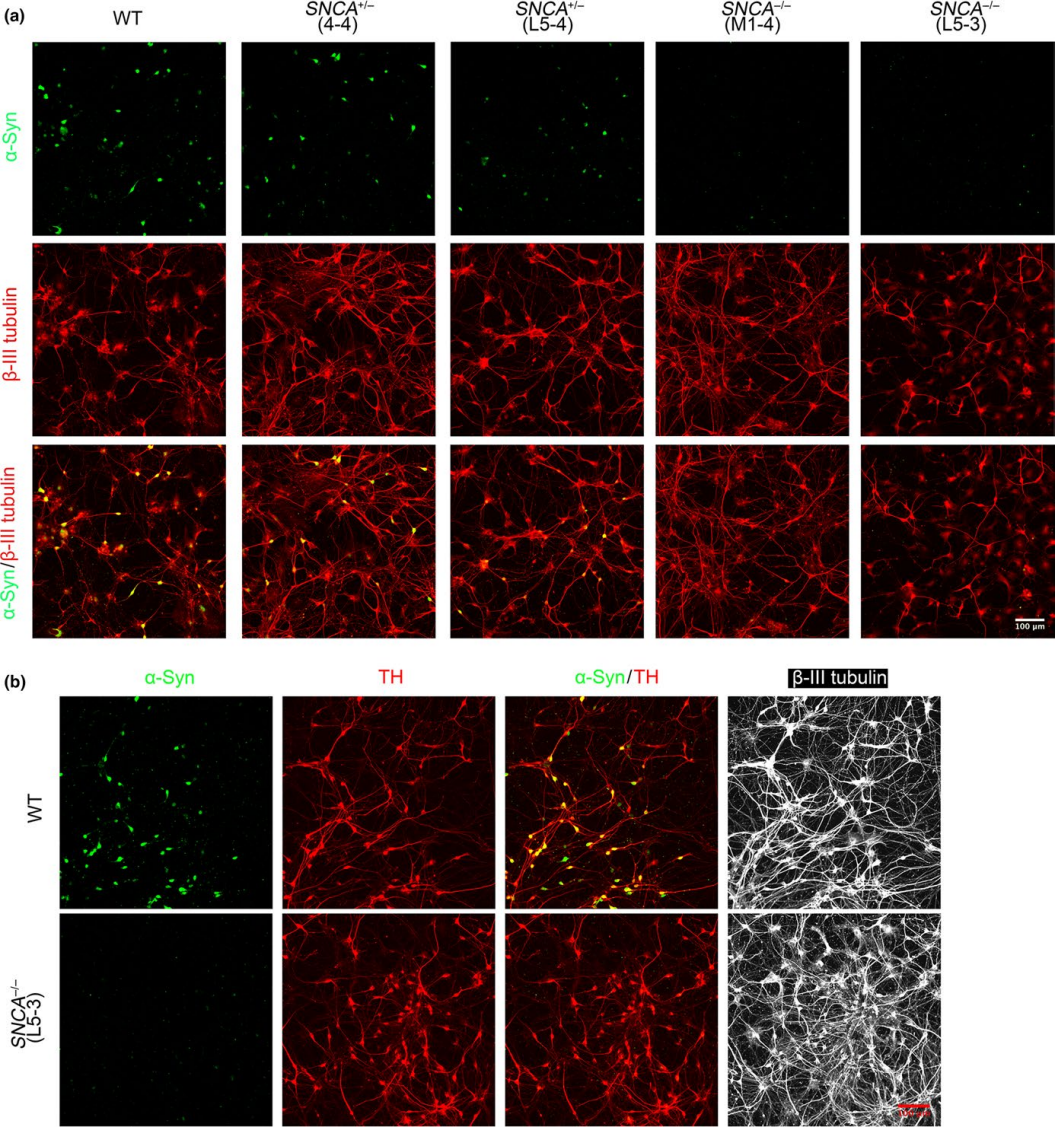
α‐Synuclein protein is absent from *SNCA* ^−/−^ mDA neurons and reduced in *SNCA* ^+/−^ clones. (a) Immunostaining of day 44 mDA neurons differentiated from *SNCA* ^+/−^, *SNCA* ^−/−^ or WT hESCs; total α‐synuclein (green)/β‐III tubulin (red). (b) Immunostaining of day 44 mDA neurons differentiated from *SNCA* ^−/−^ or WT hESCs: stained with total α‐synuclein (green)/TH (red)/β‐III tubulin (white)

Reduction or deletion of the *SNCA* gene may affect differentiation efficiency into mDA neurons. In order to address this question, we differentiated two *SNCA* ^+/−^ hESC lines and two *SNCA* ^−/−^ lines, as well as the wild‐type RC17 parental hESC line, using a modified version of an established mDA neuronal protocol (Figure 2a; Nolbrant, Heuer, Parmar, & Kirkeby, 2017). FACS analysis for the floor plate marker, CORIN, at day 16 of differentiation showed that all cell lines produced a highly pure population of floor plate cells regardless of *SNCA* genotype (Figure 2b). Gene expression analysis at day 44 of differentiation showed a down‐regulation of the pluripotent markers *OCT4* and *NANOG* in all genotypes of cell line, and an up‐regulation of the dopaminergic markers *NURR1*,* SOX6*,* DAT*, and *VMAT2* in all cell lines regardless of *SNCA* genotype (Figure 2c). This suggests deletion of the *SNCA* gene does not have a major effect on the differentiation of hESCs into mDA neurons. Immunostaining for the pan‐neuronal marker, β‐III tubulin, the dopaminergic enzyme tyrosine hydroxylase (TH), and the midbrain marker, FOXA2, further confirmed there were no gross differences in the differentiation potential of *SNCA* ^−/−^ hESCs to produce dopaminergic neurons (Figure 3a,b). Wild‐type and both *SNCA* ^+/−^ hESC lines exhibited nuclear and cytoplasmic α‐synuclein expression, while mDA neurons differentiated from both *SNCA* ^−/−^ cell lines lacked this expression (Figure 4), in agreement with the western blotting data. The localization of α‐synuclein is not synaptic in these neuronal cultures at day 44 because they were not sufficiently mature yet. This is in agreement with the observation that α‐synuclein is not redistributed from the cell body to synapses until 18 weeks of gestation (Galvin, Schuck, Lee, & Trojanowski, 2001).

### 3.3 α‐Synuclein‐null mDA neurons are resistant to acquiring Lewy‐like pathology

**Figure 5:**
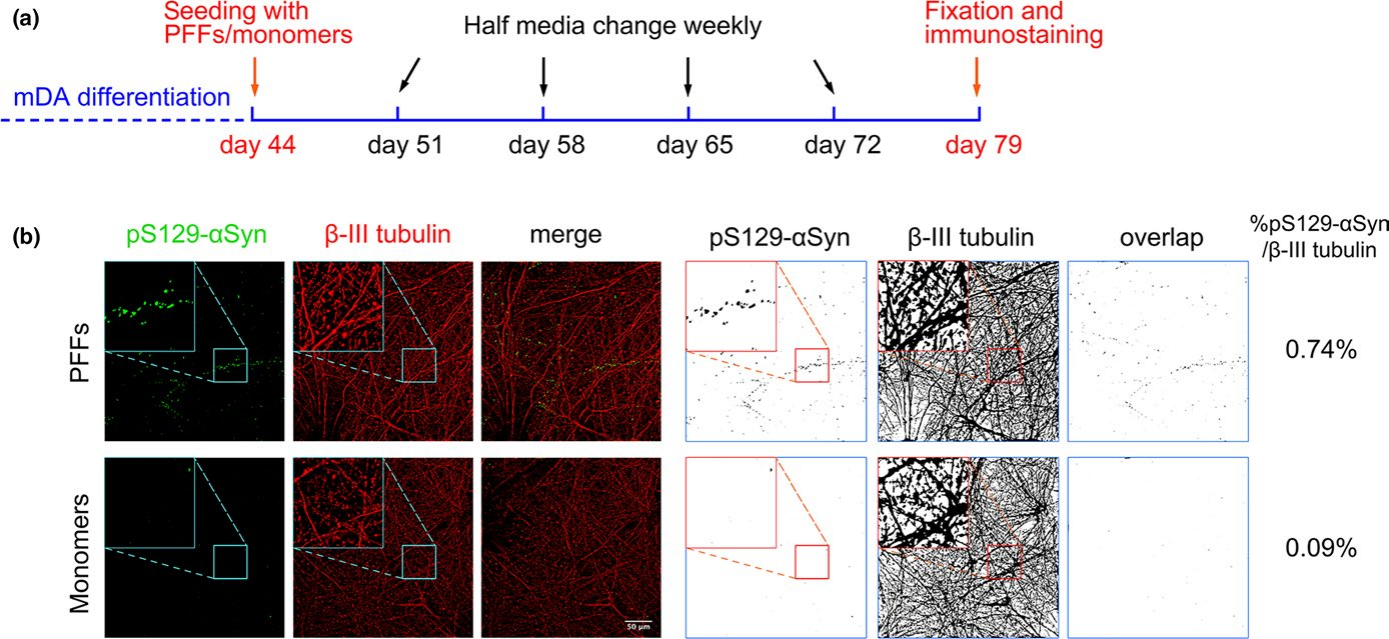
α‐Synuclein pre‐formed fibrils (PFFs) seeding and the process of phosphorylated Serine 129 α‐synuclein (pS129‐αSyn) quantification. (a) Time line of PFFs seeding and pS129‐αSyn quantification during mDA differentiation. (b) WT mDA neurons seeded with PFFs for 5 weeks, immunostained with pS129‐αSyn (green) and β‐III tubulin (red): colour images (left panel) and respective binary images (right panel), with partially 2.8× enlarged images and percentage of overlapping pS129‐αSyn staining normalized to β‐III tubulin immunostaining

We induced synucleinopathy in human neurons by using an established α‐synuclein preformed fibrils (PFFs) model (Volpicelli‐Daley et al., 2011). Human α‐synuclein protein was produced using a HEK 293 mammalian expression system. Monomeric protein was agitated for 10 days to form fibrils that were quality controlled by JC‐1 fluorescence (Lee et al., 2009), and Fourier‐transform infrared (FTIR) spectrometry analysis (Kaylor et al., 2005) (Supporting Information Figure S5). The fibrillar protein was then sonicated on ice to produce PFFs. Recombinant α‐synuclein PFFs, or monomers, were added to the culture medium of day 44 mDA neurons at a concentration of 5 μg/ml. After 5 weeks of additional culture and maturation of neurons, the cells were immunostained for β‐III tubulin and phosphoSer‐129‐α‐synuclein (pS129‐αSyn) (Figure 5a), a posttranslational modification that occurs in Lewy bodies and is a reliable biomarker of aggregated α‐synuclein (Fujiwara et al., 2002). mDA neurons exposed to α‐synuclein PFFs exhibited numerous pS129‐αSyn structures within neurons after 5 weeks, while α‐synuclein monomers were unable to induce the formation of these structures (Figure 5b). To quantify the extent of Lewy‐like molecular pathology in neurons, the immunostaining of pS129‐αSyn and β‐III tubulin were converted to binary images and the percentage of β‐III tubulin‐positive space (i.e. neurons) occupied by pS129‐αSyn signal was calculated (Figure 5b).

**Figure 6:**
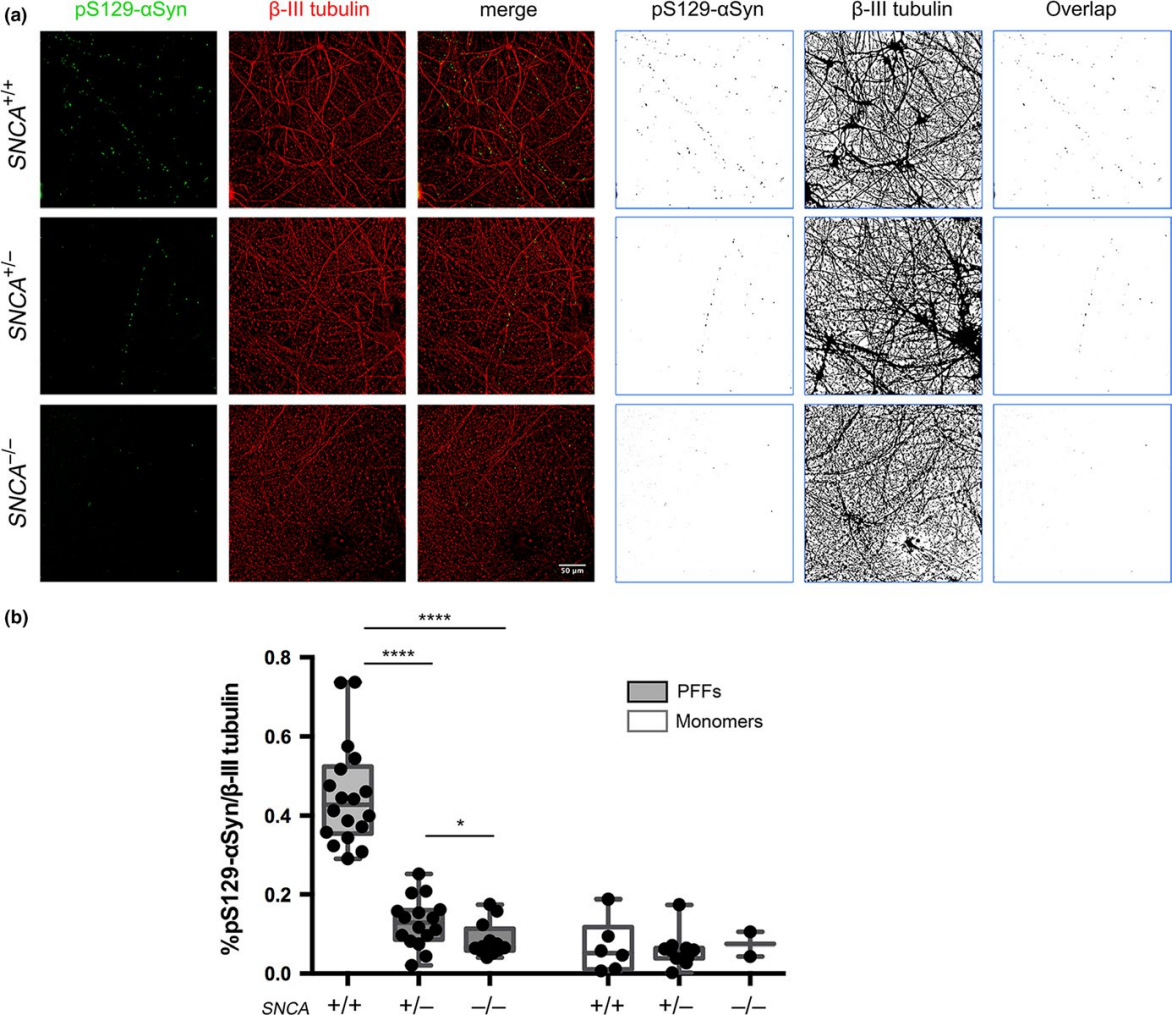
pS129‐αSyn quantification of *SNCA* ^+/−^, *SNCA* ^−/−^ or WT mDA neurons seeded with PFFs or monomers. (a) *SNCA* ^+/−^, *SNCA* ^−/−^ and WT mDA neurons seeded with PFFs for 5 weeks, immunostained with pS129‐αSyn (green) and β‐III tubulin (red): colour images (left panel) and respective binary images (right panel). (b) Quantification of overlapping pS129‐αSyn immunostaining in PFF and monomer‐seeded mDA neurons normalized to β‐III tubulin immunostaining presented as box plots. (PFF data: *SNCA* ^+/+^, *n* = 6, 18 images; *SNCA* ^+/−^, *n* = 6, 16 images; *SNCA* ^−/−^, *n* = 4, 12 images. Monomer data: *SNCA* ^+/+^, *n* = 3, 6 images; *SNCA* ^+/−^, *n* = 4, 11 images; *SNCA* ^−/−^, *n* = 1, 2 images) ****p < 0.0001, *p < 0.05: Mann–Whitney *U* test

**Figure 7:**
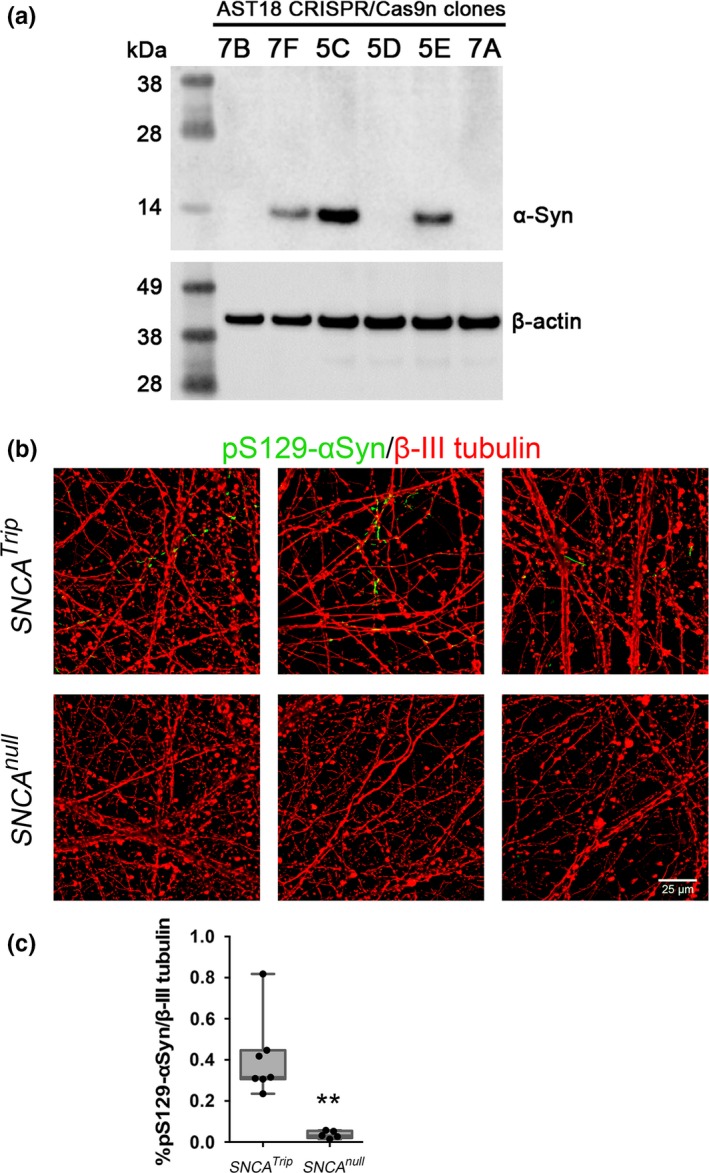
pS129‐αSyn pathology model is reproducible in hiPSCs. (a) Western blotting of triplication *SNCA* AST18 iPSC clones targeted for deletion of exon 2 of *SNCA* gene. (b) *SNCA*^T^ ^rip^ and *SNCA* ^null^ (7B clone) mDA neurons seeded with α‐Syn PFFs or monomers, immunostained with pS129‐αSyn (green) and β‐III tubulin (red). Three representative images are shown for each condition. (c) Quantification of pS129‐αSyn immunostaining in β‐III tubulin‐positive domain for PFF and monomer‐seeded mDA neurons presented as box plots. **p < 0.01: Mann–Whitney *U* test

We then compared wild‐type, *SNCA* ^+/−^, and *SNCA* ^−/−^ mDA neurons for their susceptibility to form phosphorylated α‐synuclein structures in response to pathology‐inducing PFFs in the same time‐frame (5 weeks). Wild‐type mDA neurons exhibited extensive pS129‐αSyn structures due to α‐synuclein PFF seeding (Figure 6a). Heterozygous *SNCA* ^+/−^ mDA neurons exhibited much less pS129‐αSyn immunostaining, but a small number of neurons showed punctate pS129‐αSyn‐positive structures along axons (Figure 6a). Knock‐out *SNCA* ^−/−^ mDA neurons did not possess any pS129‐αSyn‐positive structures that followed axonal tracks or co‐localised with β‐III tubulin‐positive neuronal cell bodies (Figure 6a). Quantification of the pS129‐αSyn structures with respect to the area of β‐III tubulin‐positive neurons showed that *SNCA* ^+/−^ and *SNCA* ^−/−^ mDA neurons were significantly less susceptible to forming Lewy‐like structures than wild‐type neurons when challenged with α‐synuclein PFFs (Figure 6b). Control monomeric α‐synuclein at the same concentration or vehicle, PBS, were unable to initiate the formation of pS129‐αSyn structures in any of the mDA neuronal cultures (Figure 6b, Supporting Information Figure S6). To confirm these results with an independent cell line, we used the same strategy to target exon 2 of the *SNCA* gene in AST18 iPSCs, which harbour a triplication containing the *SNCA* locus (Devine et al., 2011). We obtained four clonal iPSC lines that lacked α‐synuclein protein expression based on western blotting (Figure 7a). AST18 (*SNCA* ^Trip^) and AST18‐7B (*SNCA* ^null^) iPSCs were differentiated into mDA neurons and treated with α‐synuclein PFFs using the same conditions for hESC‐derived neurons. Upon fixing and immunostaining for pS129‐αSyn it was observed that *SNCA* ^Trip^ neurons exhibited numerous pS129‐αSyn structures along neurites, while these structures were virtually absent in the *SNCA* ^null^ neurons (Figure 7b). Image quantification of the pS129‐αSyn area demonstrated a significant reduction of the Lewy‐like structures in *SNCA* ^null^ neurons (Figure 7c), which is in agreement with the observation in hESC‐derived neurons.

## 4 DISCUSSION

A dopaminergic cell replacement therapy for Parkinson's is rapidly approaching clinical trials (Barker et al., 2017). We know from clinical trials with fetal mesencephalic grafts that significant dopaminergic re‐innervation can be achieved, and this is accompanied by significant clinical benefit (Lindvall et al., 1990, 1994; Ma et al., 2010). However, the cells transplanted into the Parkinson's striatum are susceptible to synucleinopathy and the formation of Lewy bodies (Kordower et al., 2008; Li et al., 2008; Mendez et al., 2008). Over time this reduces the clinical benefit of the graft and a re‐emergence of motor symptoms (Li et al., 2016). The on‐going synucleinopathy to other parts of the brain, such as the cortex, will likely contribute to a worsening of symptoms as well. Here, we have engineered human pluripotent stem cells with CRISPR/Cas9 nickase technology to reduce or remove the *SNCA* alleles encoding for α‐synuclein. Using Cas9n with four gRNAs, we were able to successfully remove the first coding exon, exon 2, of the human *SNCA* gene in clinical‐grade RC17 hESCs to produce a number of *SNCA* ^+/−^ and *SNCA* ^−/−^ clonal cell lines, as well as deleting all four alleles of *SNCA* from the α‐synuclein triplication iPSC line, AST18. All genotypes of cell line efficiently produced mDA neurons in a modified floor plate protocol suggesting that removal of the α‐synuclein protein does not grossly affect the differentiation propensity of pluripotent stem cells into this neuronal subtype. More detailed transcriptomic analysis and functional studies of dopamine release, for example, will be required to determine if more subtle differences exist between wild‐type human neurons and the ones that lack α‐synuclein, especially since *Snca* ^−/−^ mice have reduced dopamine content in the striatum despite normal numbers of dopaminergic neurons in the *substantia nigra* (Abeliovich et al., 2000; Al‐Wandi et al., 2010). Analysis of synaptic vesicle mobilisation in mature mDA neurons will also be important, since mice lacking α‐synuclein exhibit a deficit in replenishing pools of presynaptic vesicles after depletion in hippocampal neurons (Cabin et al., 2002), and this phenotype was also observed in the striatum of triple knock‐out mice lacking all three synuclein genes, *Snca*,* Sncb*,* Sncg* (Anwar et al., 2011). It is interesting to note that *Snca* ^−/−^ mice exhibit a degree of resistance to the mitochondrial toxin MPTP (Dauer et al., 2002; Robertson et al., 2004), suggesting α‐synuclein‐null grafts may have some protection against environmental toxins. However, *Snca* ^−/−^ mice are highly susceptible to neuroinvasive viruses, such as West Nile virus, while heterozygous *Snca* ^+/−^ mice do not display this phenotype (Beatman et al., 2015).

In agreement with our data, the loss of α‐synuclein in mice confers complete resistance to experimentally induced synucleinopathy (Luk et al., 2012), which is analogous to the finding that the endogenous *PrP* gene is critical to be susceptible to prion infection (Mallucci et al., 2003). When we challenged wild‐type, *SNCA* ^+/−^ and *SNCA* ^−/−^ mDA neurons with recombinant α‐synuclein PFFs, we observed a significant number of punctate pS129‐αSyn structures in wild‐type neurons, but very few or none in *SNCA* ^+/−^ and *SNCA* ^−/−^ neurons. Exposing mDA neurons to monomeric α‐synuclein protein at the same concentration was unable to produce pS129‐αSyn structures. The lack of pS129‐αSyn structures in *SNCA* ^−/−^ neurons was expected from the data published in rodent neurons and mice (Luk et al., 2012; Volpicelli‐Daley et al., 2011). However, the significantly reduced pS129‐αSyn structures in heterozygous *SNCA* ^+/−^ neurons were somewhat surprising. This is most likely due to the length of time of the assay (5 weeks). Since these neurons express about half the level of α‐synuclein protein, they would require longer time periods to accumulate and mature the pS129‐αSyn structures. This has implications for the longevity of heterozygous *SNCA* ^+/−^ grafts in patients, given the time (>10 years) it took wild‐type fetal grafts to acquire Lewy pathology and begin to fail. Reducing the α‐synuclein protein level by 50% could significantly increase the functional longevity of the graft by slowing and reducing Lewy body burden, while avoiding the potential issues of synaptic mobilisation defects or increased susceptibility to neuroinvasive viruses. Preclinical testing of *SNCA* ^+/−^ and *SNCA* ^−/−^ mDA cells will require an animal model of host‐to‐graft α‐synuclein spread. A 6‐hydroxydopamine (6‐OHDA) lesion model with AAV6‐human α‐synuclein administration in the rat has been reported (Kordower et al., 2011). Wild‐type rat ventral mesencephalon (VM) was used to graft the animals, and between 2% to 15% of the dopaminergic neurons acquired human α‐synuclein from the host (Kordower et al., 2011). A mouse model of host‐to‐graft spread has also been reported where embryonic mouse VM is transplanted on transgenic mice expressing human α‐synuclein driven by the mouse *SNCA* promoter (Hansen et al., 2011). Demonstration of disease‐resistance of human *SNCA* ^+/−^ and *SNCA* ^−/−^ mDA cells in one of these models, or similar models, will be required prior to clinical translation.

Genome editing with CRISPR/Cas9 technology is being applied to address diverse medical problems, including cancer, HIV infection, and monogenic diseases (Hsu, Lander, & Zhang, 2014; Knott & Doudna, 2018). Genome‐edited human pluripotent stem cells for clinical trials have yet to be reported, but is set to follow the work of genome editing of other cell types, such as T cells, for medical applications. The use of Cas9 nickase technology (Ran et al., 2013), or next‐generation Cas9 enzymes (Kleinstiver et al., 2016), have significantly reduced or eliminated off‐target disruption of the genome and dramatically improved the safety profile of genome‐engineered cells for clinical applications.

Here, we have shown that *SNCA* ^+/−^ and *SNCA* ^−/−^ mDA neurons can be generated by a straight‐forward genome engineering method via hESCs or iPSCs, and they present a significant opportunity to produce next‐generation cell products that exhibit resistance to Parkinson's pathology.

## CONFLICT OF INTEREST

M.K., T.B. and P.D. are employees of UCB Biopharma. The other authors declare no conflict of interest.

## AUTHORS’ CONTRIBUTION

T.K. designed the study and wrote the paper. Y.C. and K.S.D. performed experiments and data analysis. M.K., N.J.D., M.A.C. and A.N. provided reagents, optimised protocols and assisted in data analysis. T.B., P.D. and S.R. contributed to study design and data analysis. All authors edited and approved the manuscript.

## Data Availability

All raw data for graphs in Figures 2, 6, and 7 can obtained from LabArchives.com at https://doi.org/10.25833/8bj6-e156. All other requests for data should be directed to Tilo Kunath.

## References

[ejn14286-bib-0001] Abeliovich, A. , Schmitz, Y. , Fariñas, I. , Choi‐Lundberg, D. , Ho, W. H. , Castillo, P. E. , … Rosenthal, A. (2000). Mice lacking alpha‐synuclein display functional deficits in the nigrostriatal dopamine system. Neuron, 25, 239–252. 10.1016/S0896-6273(00)80886-7 10707987

[ejn14286-bib-0002] Al‐Wandi, A. , Ninkina, N. , Millership, S. , Williamson, S. J. , Jones, P. A. , & Buchman, V. L. (2010). Absence of α‐synuclein affects dopamine metabolism and synaptic markers in the striatum of aging mice. Neurobiology of Aging, 31, 796–804. 10.1016/j.neurobiolaging.2008.11.001 19097673 PMC3146702

[ejn14286-bib-0003] Anwar, S. , Peters, O. , Millership, S. , Ninkina, N. , Doig, N. , Connor‐Robson, N. , … Buchman, V. L. (2011). Functional alterations to the nigrostriatal system in mice lacking all three members of the synuclein family. Journal of Neuroscience, 31, 7264–7274. 10.1523/JNEUROSCI.6194-10.2011 21593311 PMC3154647

[ejn14286-bib-0004] Barker, R. A. , Parmar, M. , Studer, L. , & Takahashi, J. (2017). Human trials of stem cell‐derived dopamine neurons for Parkinson's disease: Dawn of a new era. Cell Stem Cell, 21, 569–573. 10.1016/j.stem.2017.09.014 29100010

[ejn14286-bib-0005] Beatman, E. L. , Massey, A. , Shives, K. D. , Burrack, K. S. , Chamanian, M. , Morrison, T. E. , & Beckham, J. D. (2015). Alpha‐synuclein expression restricts RNA viral infections in the brain. Journal of Virology, 90, 2767–2782. 26719256 10.1128/JVI.02949-15 PMC4810656

[ejn14286-bib-0006] Braak, H. , Ghebremedhin, E. , Rüb, U. , Bratzke, H. , & Del Tredici, K. (2004). Stages in the development of Parkinson's disease‐related pathology. Cell and Tissue Research, 318, 121–134. 10.1007/s00441-004-0956-9 15338272

[ejn14286-bib-0007] Cabin, D. E. , Shimazu, K. , Murphy, D. , Cole, N. B. , Gottschalk, W. , McIlwain, K. L. , … Nussbaum, R. L. (2002). Synaptic vesicle depletion correlates with attenuated synaptic responses to prolonged repetitive stimulation in mice lacking alpha‐synuclein. Journal of Neuroscience, 22, 8797–8807. 10.1523/JNEUROSCI.22-20-08797.2002 12388586 PMC6757677

[ejn14286-bib-0008] Chu, Y. , & Kordower, J. H. (2010). Lewy body pathology in fetal grafts. Annals of the New York Academy of Sciences, 1184, 55–67. 10.1111/j.1749-6632.2009.05229.x 20146690

[ejn14286-bib-0009] Dauer, W. , Kholodilov, N. , Vila, M. , Trillat, A.‐C. , Goodchild, R. , Larsen, K. E. , … Hen, R. (2002). Resistance of α‐synuclein null mice to the parkinsonian neurotoxin MPTP. Proceedings of the National Academy of Sciences of the United States of America, 99, 14524–14529. 10.1073/pnas.172514599 12376616 PMC137916

[ejn14286-bib-0010] De Sousa, P. A. , Tye, B. J. , Bruce, K. , Dand, P. , Russell, G. , Collins, D. M. , … Courtney, A. (2016). Derivation of the clinical grade human embryonic stem cell line RCe021‐A (RC‐17). Stem Cell Research, 17, 1–5. 10.1016/j.scr.2016.04.019 27558596

[ejn14286-bib-0011] Devine, M. J. , Ryten, M. , Vodicka, P. , Thomson, A. J. , Burdon, T. , Houlden, H. , … Kunath, T. (2011). Parkinson's disease induced pluripotent stem cells with triplication of the α‐synuclein locus. Nature Communications, 2, 440 10.1038/ncomms1453 PMC3265381 21863007

[ejn14286-bib-0012] Farrer, M. , Kachergus, J. , Forno, L. , Lincoln, S. , Wang, D.‐S. , Hulihan, M. , … Langston, J. W. (2004). Comparison of kindreds with Parkinsonism and alpha‐synuclein genomic multiplications. Annals of Neurology, 55, 174–179. 10.1002/(ISSN)1531-8249 14755720

[ejn14286-bib-0013] Fujiwara, H. , Hasegawa, M. , Dohmae, N. , Kawashima, A. , Masliah, E. , Goldberg, M. S. , … Iwatsubo, T. (2002). Alpha‐Synuclein is phosphorylated in synucleinopathy lesions. Nature Cell Biology, 4, 160–164. 10.1038/ncb748 11813001

[ejn14286-bib-0014] Galvin, J. E. , Schuck, T. M. , Lee, V. M. Y. , & Trojanowski, J. Q. (2001). Differential expression and distribution of α‐, β‐, and γ‐synuclein in the developing human substantia Nigra. Experimental Neurology, 168, 347–355. 10.1006/exnr.2000.7615 11259122

[ejn14286-bib-0015] Hagell, P. , Piccini, P. , Björklund, A. , Brundin, P. , Rehncrona, S. , Widner, H. , … Lindvall, O. (2002). Dyskinesias following neural transplantation in Parkinson's disease. Nature Neuroscience, 5, 627–628. 10.1038/nn863 12042822

[ejn14286-bib-0016] Hansen, C. , Angot, E. , Bergström, A.‐L. , Steiner, J. A. , Pieri, L. , Paul, G. , … Brundin, P. (2011). α‐Synuclein propagates from mouse brain to grafted dopaminergic neurons and seeds aggregation in cultured human cells. The Journal of Clinical Investigation, 121, 715–725. 10.1172/JCI43366 21245577 PMC3026723

[ejn14286-bib-0017] Hsu, P. D. , Lander, E. S. , & Zhang, F. (2014). Development and applications of CRISPR‐Cas9 for genome engineering. Cell, 157, 1262–1278. 10.1016/j.cell.2014.05.010 24906146 PMC4343198

[ejn14286-bib-0018] Kaylor, J. , Bodner, N. , Edridge, S. , Yamin, G. , Hong, D.‐P. , & Fink, A. L. (2005). Characterization of oligomeric intermediates in alpha‐synuclein fibrillation: FRET studies of Y125W/Y133F/Y136F alpha‐synuclein. Journal of Molecular Biology, 353, 357–372. 10.1016/j.jmb.2005.08.046 16171820

[ejn14286-bib-0019] Kirkeby, A. , Nolbrant, S. , Tiklova, K. , Heuer, A. , Kee, N. , Cardoso, T. , … Parmar, M. (2017). Predictive markers guide differentiation to improve graft outcome in clinical translation of hESC‐based therapy for Parkinson's disease. Cell Stem Cell, 20, 135–148. 10.1016/j.stem.2016.09.004 28094017 PMC5222722

[ejn14286-bib-0020] Kleinstiver, B. P. , Pattanayak, V. , Prew, M. S. , Tsai, S. Q. , Nguyen, N. T. , Zheng, Z. , & Joung, J. K. (2016). High‐fidelity CRISPR‐Cas9 nucleases with no detectable genome‐wide off‐target effects. Nature, 529, 490–495. 10.1038/nature16526 26735016 PMC4851738

[ejn14286-bib-0021] Knott, G. J. , & Doudna, J. A. (2018). CRISPR‐Cas guides the future of genetic engineering. Science, 361, 866–869. 10.1126/science.aat5011 30166482 PMC6455913

[ejn14286-bib-0022] Kordower, J. H. , Chu, Y. , Hauser, R. A. , Freeman, T. B. , & Olanow, C. W. (2008). Lewy body‐like pathology in long‐term embryonic nigral transplants in Parkinson's disease. Nature Medicine, 14, 504–506. 10.1038/nm1747 18391962

[ejn14286-bib-0023] Kordower, J. H. , Dodiya, H. B. , Kordower, A. M. , Terpstra, B. , Paumier, K. , Madhavan, L. , … Collier, T. J. (2011). Transfer of host‐derived alpha synuclein to grafted dopaminergic neurons in rat. Neurobiology of Diseases, 43, 552–557. 10.1016/j.nbd.2011.05.001 PMC3430516 21600984

[ejn14286-bib-0024] Kriks, S. , Shim, J.‐W. , Piao, J. , Ganat, Y. M. , Wakeman, D. R. , Xie, Z. , … Studer, L. (2011). Dopamine neurons derived from human ES cells efficiently engraft in animal models of Parkinson's disease. Nature, 480, 547–551. 10.1038/nature10648 22056989 PMC3245796

[ejn14286-bib-0025] Lee, J.‐H. , Lee, I.‐H. , Choe, Y.‐J. , Kang, S. , Kim, H. Y. , Gai, W.‐P. , … Paik, S. R. (2009). Real‐time analysis of amyloid fibril formation of α‐synuclein using a fibrillation‐state‐specific fluorescent probe of JC‐1. The Biochemical Journal, 418, 311 10.1042/BJ20081572 19007333

[ejn14286-bib-0026] Li, J.‐Y. , Englund, E. , Holton, J. L. , Soulet, D. , Hagell, P. , Lees, A. J. , … Brundin, P. (2008). Lewy bodies in grafted neurons in subjects with Parkinson's disease suggest host‐to‐graft disease propagation. Nature Medicine, 14, 501–503. 10.1038/nm1746 18391963

[ejn14286-bib-0027] Li, W. , Englund, E. , Widner, H. , Mattsson, B. , van Westen, D. , Lätt, J. , … Li, J.‐Y. (2016). Extensive graft‐derived dopaminergic innervation is maintained 24 years after transplantation in the degenerating parkinsonian brain. Proceedings of the National Academy of Sciences of the United Statest of America, 113, 6544–6549. 10.1073/pnas.1605245113 PMC4988567 27140603

[ejn14286-bib-0028] Lindvall, O. , Brundin, P. , Widner, H. , Rehncrona, S. , Gustavii, B. , Frackowiak, R. , … Björklund, A. (1990). Grafts of fetal dopamine neurons survive and improve motor function in Parkinson's disease. Science, 247, 574–577. 10.1126/science.2105529 2105529

[ejn14286-bib-0029] Lindvall, O. , Rehncrona, S. , Gustavii, B. , Brundin, P. , Astedt, B. , Widner, H. , … Olson, L. (1988). Fetal dopamine‐rich mesencephalic grafts in Parkinson's disease. Lancet, 2, 1483–1484. 10.1016/S0140-6736(88)90950-6 2904587

[ejn14286-bib-0030] Lindvall, O. , Sawle, G. , Widner, H. , Rothwell, J. C. , Björklund, A. , Brooks, D. , … Odin, P. (1994). Evidence for long‐term survival and function of dopaminergic grafts in progressive Parkinson's disease. Annals of Neurology, 35, 172–180. 10.1002/ana.410350208 8109898

[ejn14286-bib-0031] Luk, K. C. , Kehm, V. , Carroll, J. , Zhang, B. , O'Brien, P. , Trojanowski, J. Q. , & Lee, V. M. Y. (2012). Pathological alpha‐synuclein transmission initiates parkinson‐like neurodegeneration in nontransgenic mice. Science, 338, 949–953. 10.1126/science.1227157 23161999 PMC3552321

[ejn14286-bib-0032] Ma, Y. , Tang, C. , Chaly, T. , Greene, P. , Breeze, R. , Fahn, S. , … Eidelberg, D. (2010). Dopamine cell implantation in Parkinson's disease: Long‐term clinical and (18)F‐FDOPA PET outcomes. Journal of Nuclear Medicine, 51, 7–15. 10.2967/jnumed.109.066811 20008998 PMC2946843

[ejn14286-bib-0033] Mali, P. , Yang, L. , Esvelt, K. M. , Aach, J. , Guell, M. , DiCarlo, J. E. , … Church, G. M. (2013). RNA‐guided human genome engineering via Cas9. Science, 339, 823–826. 10.1126/science.1232033 23287722 PMC3712628

[ejn14286-bib-0034] Mallucci, G. , Dickinson, A. , Linehan, J. , Klöhn, P.‐C. , Brandner, S. , & Collinge, J. (2003). Depleting neuronal PrP in prion infection prevents disease and reverses spongiosis. Science, 302, 871–874. 10.1126/science.1090187 14593181

[ejn14286-bib-0035] Mendez, I. , Viñuela, A. , Astradsson, A. , Mukhida, K. , Hallett, P. , Robertson, H. , … Isacson, O. (2008). Dopamine neurons implanted into people with Parkinson's disease survive without pathology for 14 years. Nature Medicine, 14, 507–509. 10.1038/nm1752 PMC2656682 18391961

[ejn14286-bib-0036] Neumann, J. , Bras, J. , Deas, E. , O'Sullivan, S. S. , Parkkinen, L. , Lachmann, R. H. , … Wood, N. W. (2009). Glucocerebrosidase mutations in clinical and pathologically proven Parkinson's disease. Brain, 132, 1783–1794. 10.1093/brain/awp044 19286695 PMC2702833

[ejn14286-bib-0037] Nolbrant, S. , Heuer, A. , Parmar, M. , & Kirkeby, A. (2017). Generation of high‐purity human ventral midbrain dopaminergic progenitors for in vitro maturation and intracerebral transplantation. Nature Protocols, 12, 1962–1979. 10.1038/nprot.2017.078 28858290

[ejn14286-bib-0038] Piccini, P. , Pavese, N. , Hagell, P. , Reimer, J. , Björklund, A. , Oertel, W. H. , … Lindvall, O. (2005). Factors affecting the clinical outcome after neural transplantation in Parkinson's disease. Brain, 128, 2977–2986. 10.1093/brain/awh649 16246865

[ejn14286-bib-0039] Politis, M. , Wu, K. , Loane, C. , Quinn, N. P. , Brooks, D. J. , Rehncrona, S. , … Piccini, P. (2010). Serotonergic neurons mediate dyskinesia side effects in Parkinson's patients with neural transplants. Science Translational Medicine, 2, 38ra46. 10.1126/scitranslmed.3000976 20592420

[ejn14286-bib-0040] Ran, F. A. , Hsu, P. D. , Lin, C.‐Y. , Gootenberg, J. S. , Konermann, S. , Trevino, A. E. , … Zhang, F. (2013). Double nicking by RNA‐guided CRISPR Cas9 for enhanced genome editing specificity. Cell, 154, 1380–1389. 10.1016/j.cell.2013.08.021 23992846 PMC3856256

[ejn14286-bib-0041] Robertson, D. C. , Schmidt, O. , Ninkina, N. , Jones, P. A. , Sharkey, J. , & Buchman, V. L. (2004). Developmental loss and resistance to MPTP toxicity of dopaminergic neurones in substantia nigra pars compacta of gamma‐synuclein, alpha‐synuclein and double alpha/gamma‐synuclein null mutant mice. Journal of Neurochemistry, 89, 1126–1136. 10.1111/j.1471-4159.2004.02378.x 15147505

[ejn14286-bib-0042] Spillantini, M. G. , Schmidt, M. L. , Lee, V. M. Y. , Trojanowski, J. Q. , Jakes, R. , & Goedert, M. (1997). Alpha‐synuclein in Lewy bodies. Nature, 388, 839–840. 10.1038/42166 9278044

[ejn14286-bib-0043] Volpicelli‐Daley, L. A. , Luk, K. C. , Patel, T. P. , Tanik, S. A. , Riddle, D. M. , Stieber, A. , … Lee, V. M. Y. (2011). Exogenous α‐synuclein fibrils induce lewy body pathology leading to synaptic dysfunction and neuron death. Neuron, 72, 57–71. 10.1016/j.neuron.2011.08.033 21982369 PMC3204802

