# Supplementary material for: Engineering synucleinopathy‐resistant human dopaminergic neurons by CRISPR‐mediated deletion of the SNCA gene
Source: Eur J Neurosci. 2018 Dec 21;49(4):510–24. doi: 10.1111/ejn.14286 (PMC6492083; doi:10.1111/ejn.14286)
Supplement: Supplementary file 1 [file EJN-49-510-s001.docx]

**Supplementary Information**

**Supplementary Figure S1.** A) *Eco*RI digestion of four independent TOPO-cloned PCR products from ~450bp PCR of putative *SNCA*^+/–^ or *SNCA*­^–/–^ clones. B) *Eco*RI digestion of seven independent TOPO-cloned PCR products from ~1kb PCR of clone M1-4.


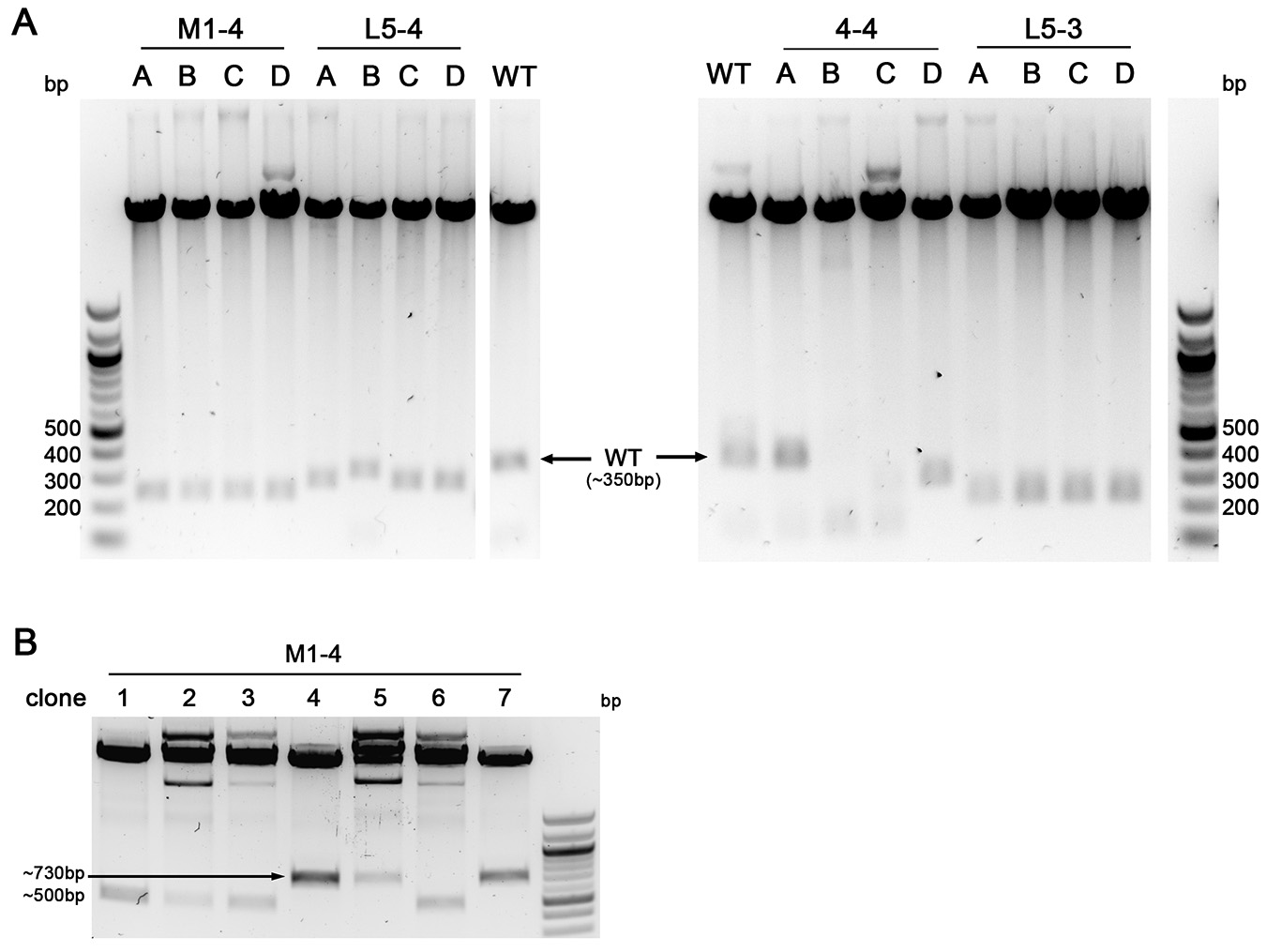


**Supplementary Figure S2.** Sequencing traces of TOPO-cloned products of *SNCA* alleles from *SNCA*^+/–^ clones 4-4 and L5-4.


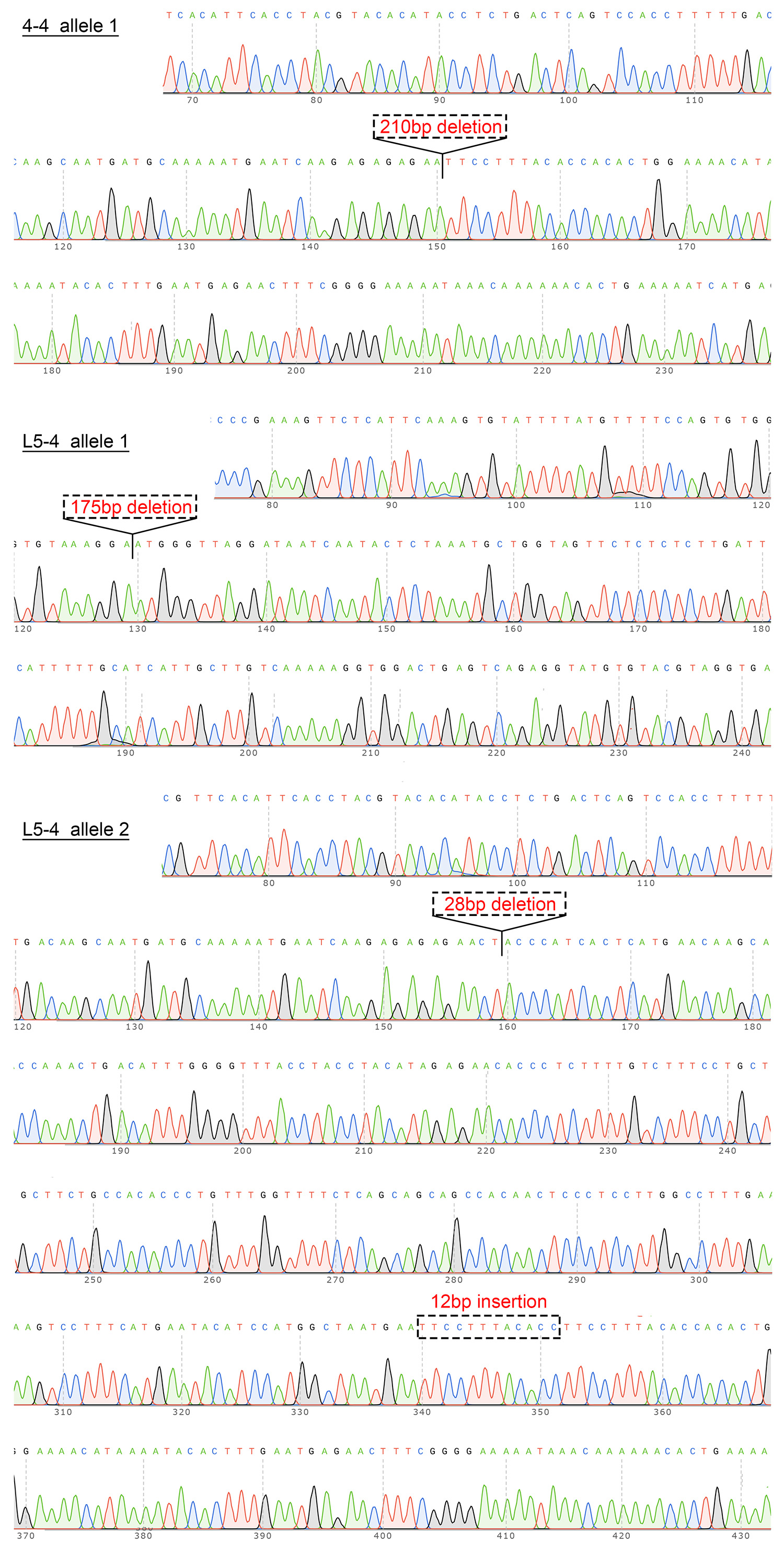


**Supplementary Figure S3.** Sequencing traces of TOPO-cloned products of *SNCA* alleles from *SNCA*­^–/–^ clone M1-4 and PCR product of *SNCA*­^–/–^ clone L5-3.


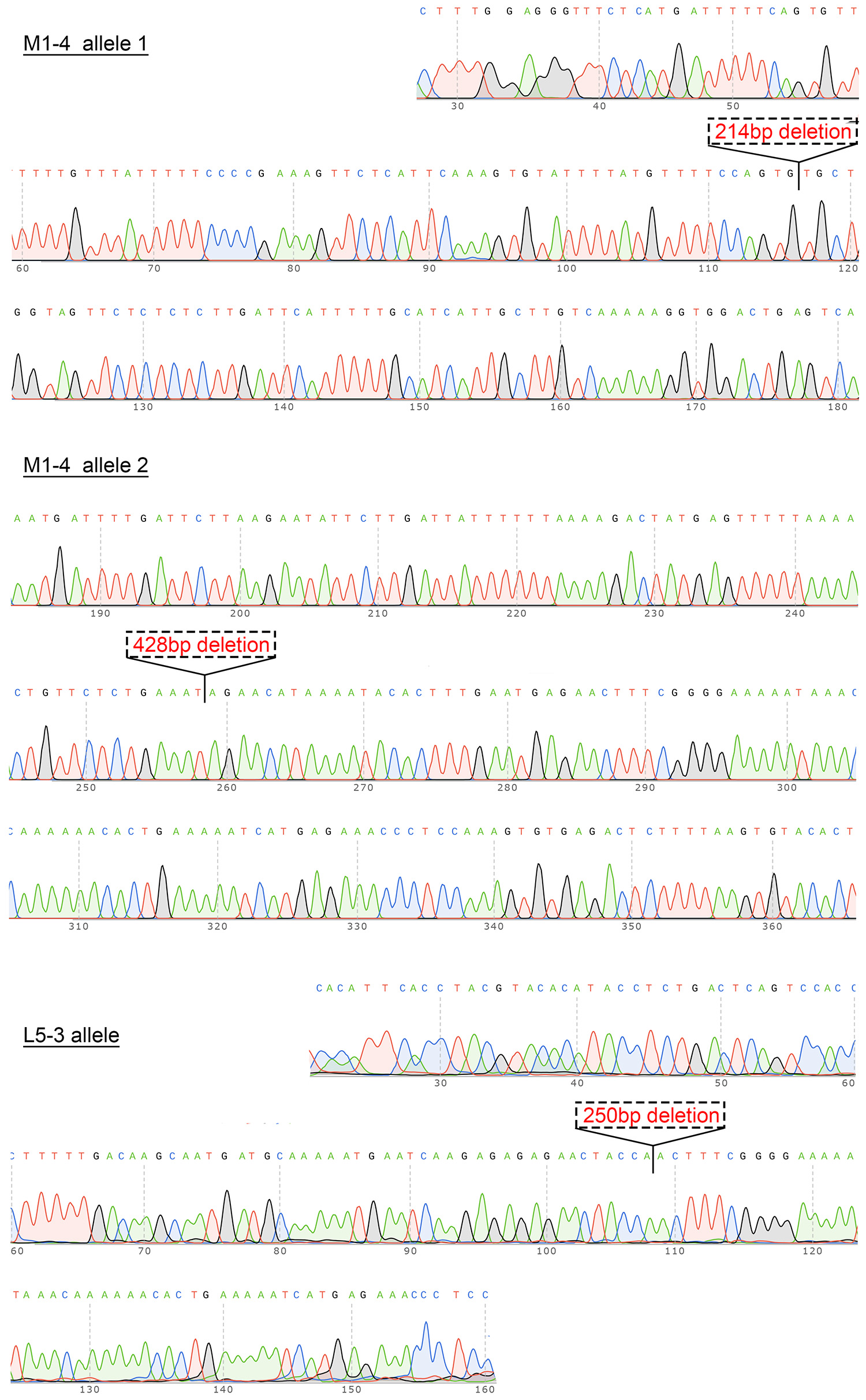


**Supplementary Figure S4.** **T7E1 assay revealed no off-target cleavage at the top 2 off-target sites of 5G1 and 3G1 gRNAs.** PCR products amplified from *SNCA*^–/–^ and *SNCA*^+/–^ clones at the top 2 off-target genomic sites of 5G1 and 3G1 gRNAs were subjected to either T7 endonuclease 1 digestion (T) or control (C) reaction: A) 5G1 off-target #1, B) 5G1 off-target #2, C) 3G1 off-target #1, and D) 3G1 off-target #2. A positive control (PC) of a targeted allele from *SNCA*^+/–^ clone 4-4 was included. Digestion products of mismatched PCR products are indicated by red arrows.


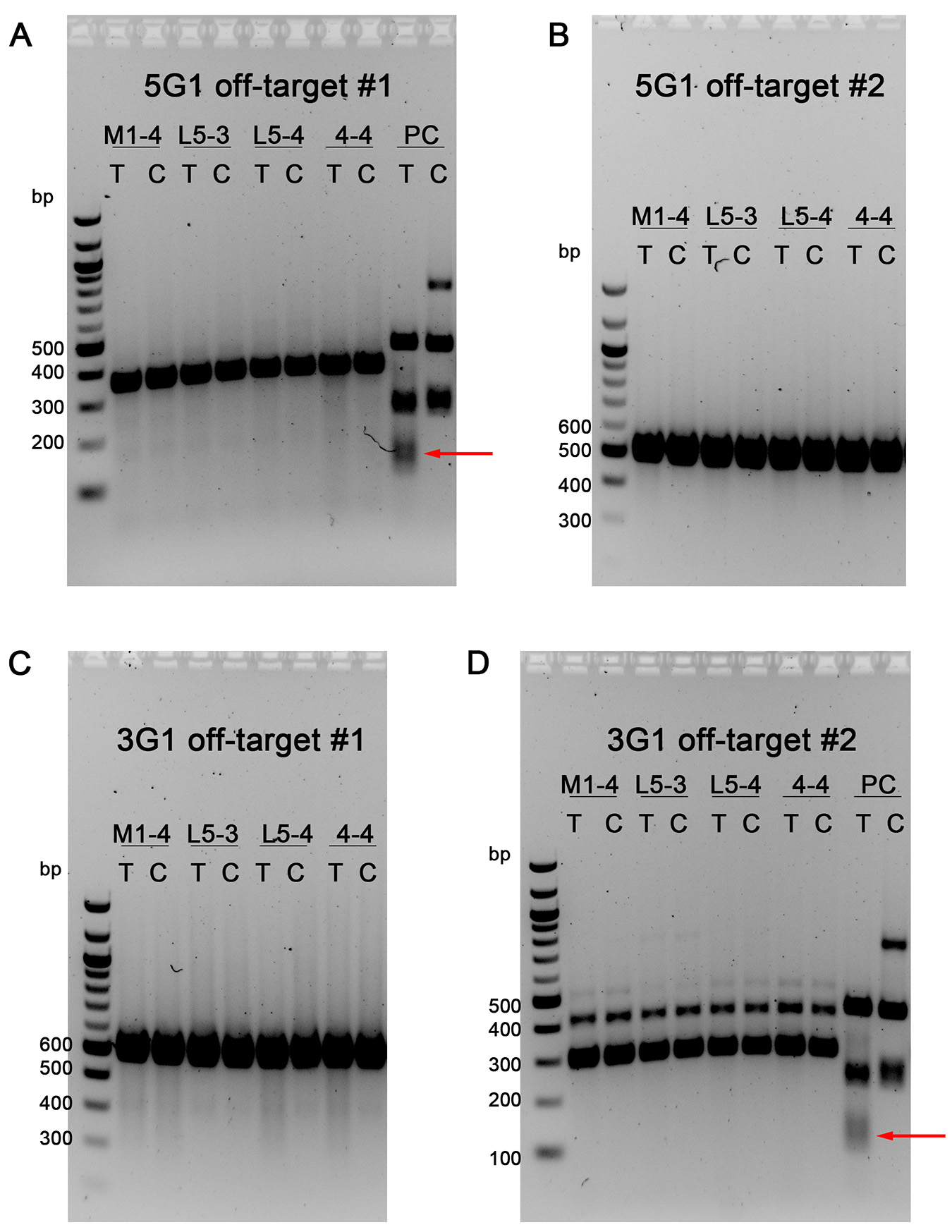


**Supplementary Figure S5. Characterisation of α-synuclein monomers and fibrils.** A**)** NuPage gel electrophoresis image of purified denatured human α-synuclein monomer. B) JC-1 fluorescence λemission = 500-600nm of α-synuclein monomer and fibrils (λmax = 540nm), λexcitation = 490nm. C) FTIR absorbance of α-synuclein monomer at 1645cm^-1^ indicative for random coil and fibril at 1627cm^-1^ indicative of β-sheet structures.


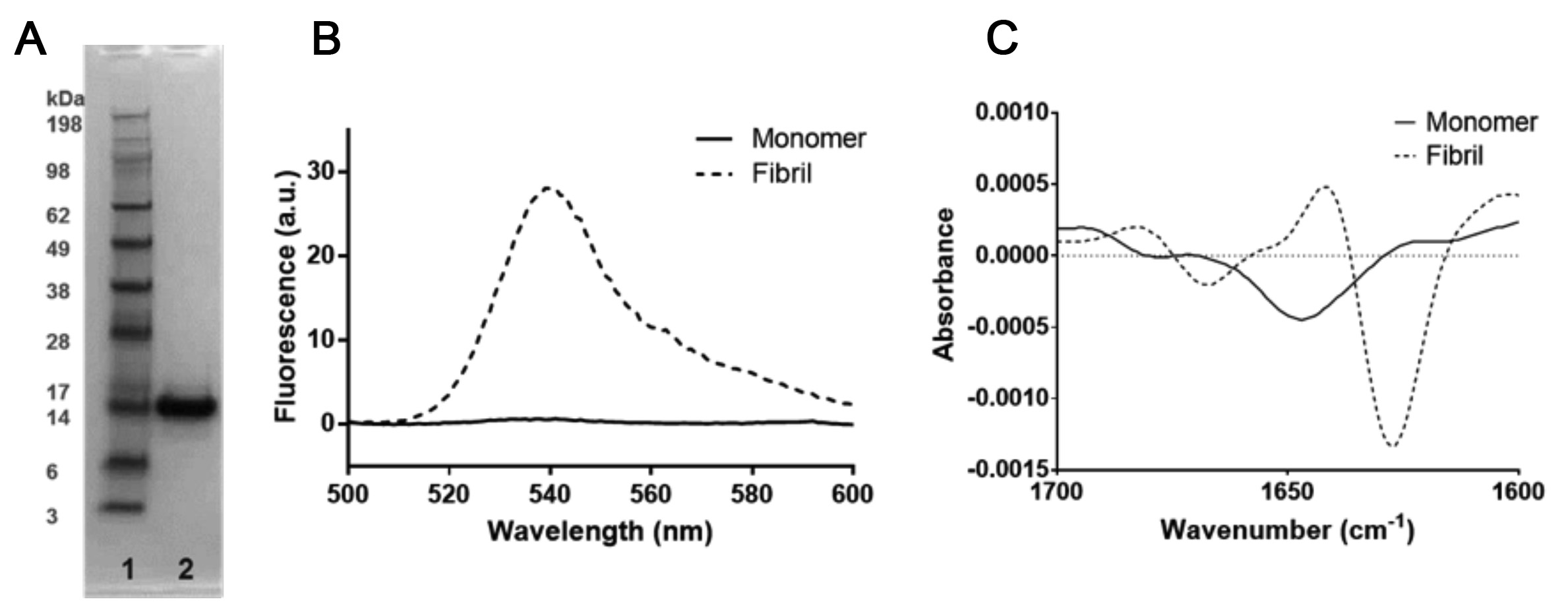


**Supplementary Figure S6. Treating mDA neurons with α-Syn monomers or vehicle control does not result in the formation of pS129-αSyn structures.** *SNCA*^+/–^, *SNCA*^–/–^ and WT mDA neurons seeded with α-Syn A) monomers or B) vehicle control, PBS, for 5 weeks, immunostained with pS129-αSyn (green) and β-III tubulin (red): colour images (left panels) and respective binary images (right panels).


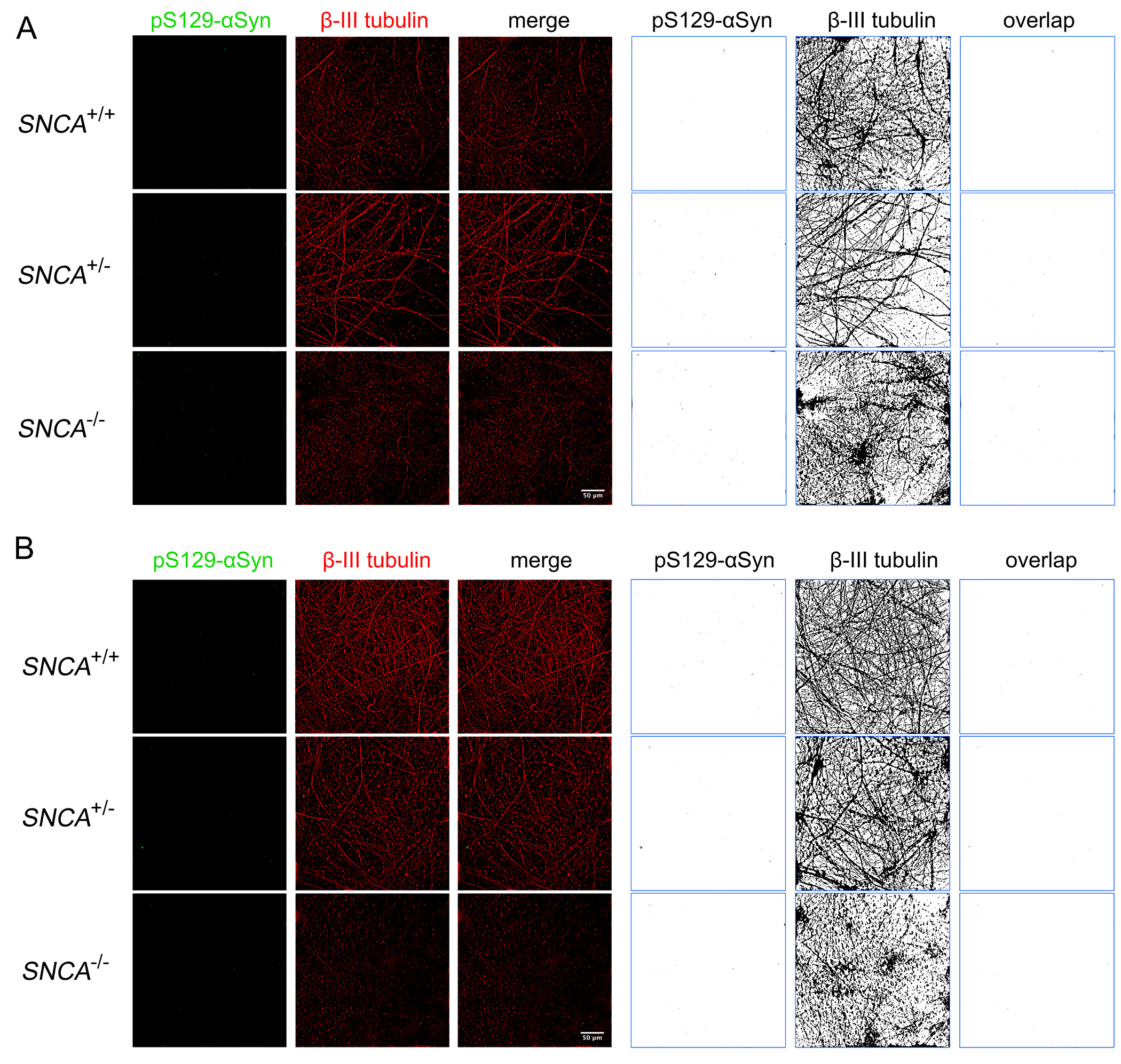


**Supplementary Table S1:** Catalogue numbers

| **Reagent** | **Company** | **Cat. No.** |
| --- | --- | --- |
| DPBS | Sigma | 14040-083 |
| Cell culture plates | Corning | 3516, 3524, 3548, 3595 |
| StemMACS iPS-Brew XF | Miltenyi Biotec | 130-107-086 |
| UltraPure 0.5 M EDTA | Thermo Fisher Scientific | 15575-038 |
| Accutase | Sigma | A6964 |
| Laminin-521 | BioLamina | LN521 |
| Laminin-111 | Biolamina | LN111 |
| DMEM/F12, no glutamine | Thermo Fisher Scientific | 21331-020 |
| Neurobasal Medium | Thermo Fisher Scientific | 21103-049 |
| B27 supplement | Thermo Fisher Scientific | 12587-010 |
| N2 supplement | Thermo Fisher Scientific | 17502-048 |
| L-Glutamine | Thermo Fisher Scientific | 25030123 |
| Y27632 | Tocris | 1254 |
| SB431542 | Millipore | 616461 |
| LDN-193189 | Miltenyi Biotec | 130-103-925 |
| Shh-C24II | R&D Systems | 1845-SH-500 |
| CHIR99021 | Miltenyi Biotec | 130-103-926 |
| FGF8b | R&D Systems | 423-F8/CF |
| heparin | Sigma | H3149 |
| ascorbic acid | Sigma | A4403 |
| brain-derived neurotrophic factor (BDNF) | Peprotech | 450-02 |
| glial cell line-derived neurotrophic factor (GDNF) | Peprotech | 450-10 |
| dibutyryl cyclic AMP | Sigma | D0627 |
| DAPT | Tocris | 2634 |
| poly-ornithine | Sigma | P4957 |
| µ-Slide 8 Well | ibidi | 80827 |
| formaldehyde solution 37-41% | Fisher | F/1501/PB08 |
| donkey serum | Sigma | D9663 |
| Triton-X 100 | Fisher | BP-151-100 |
| PBS | Thermo Fisher Scientific | 18912-014 |
| rabbit anti-tyrosine hydroxylase | Millipore | AB152 |
| goat anti-HNF-3β/FOXA2 (M20) | Santa Cruz | sc-6554 |
| mouse anti-alpha-synuclein [Syn 204] | Abcam | ab3309 |
| mouse anti-α-synuclein | BD Biosciences | 610787 |
| rabbit anti-phospho S129 α-synuclein | Abcam | A51253 |
| mouse anti-β-III tubulin | Abcam | T8660 |
| rat anti-CORIN | R&D Systems | MAB2209 |
| mouse anti-β-actin antibody, HRP conjugate | Abcam | ab20272 |
| donkey anti-rabbit Alexa Fluor-488 | Thermo Fisher Scientific | A21206 |
| donkey anti-goat Alexa Fluor-568 | Thermo Fisher Scientific | A11057 |
| donkey anti-mouse Alexa Fluor-647 | Abcam | Ab150107 |
| donkey anti-rat IgG Alexa Fluor-488 | Thermo Fisher Scientific | A21208 |
| goat anti-mouse IgG2a Alexa Fluor-488 | Thermo Fisher Scientific | A21131 |
| goat anti-mouse IgG1 Alexa Fluor-488 | Thermo Fisher Scientific | A21121 |
| goat anti-mouse IgG2b Alexa Fluor-647 | Thermo Fisher Scientific | A21242 |
| Anti-Mouse IgG (H+L), HRP conjugate | Promega | W4028 |
| DAPI | Thermo Fisher Scientific | D1306 |
| fetal calf serum | Thermo Fisher Scientific | 10270-106 |
| Amaxa™ Human Stem Cell Nucleofector™ Kit 1 | Lonza | VPH-5012 |
| Matrigel (Geltrex™ LDEV-Free, hESC-Qualified, Reduced Growth Factor Basement Membrane Matrix) | Thermo Fisher Scientific | A1413301 |
| Q5® High-Fidelity DNA Polymerase | New England Biolabs | M0491 |
| T7 endonuclease I | New England Biolabs | M0302 |
| NEBuffer™ 2 | New England Biolabs | B7002 |
| UltraPure™ Agarose | Thermo Fisher Scientific | 16500-500 |
| Quick-Load® 100 bp DNA ladder | New England Biolabs | N0467 |
| 1kb DNA ladder | New England Biolabs | N3232 |
| MasterPure™ Complete DNA and RNA Purification Kit | Epicentre | MC85200 |
| SuperScript™ IV Reverse Transcriptase | Thermo Fisher Scientific | 18090010 |
| LightCycler® 480 Probes Master mix | Roche | 04707494001 |
| RIPA Lysis Buffer System | Santa Cruz | sc-24948 |
| Pierce™ BCA protein assay kit | Thermo Fisher Scientific | 23227 |
| NuPAGE™ LDS loading dye (4x) | Thermo Fisher Scientific | NP0007 |
| NuPAGE™ Sample Reducing Agent (10x) (DTT) | Thermo Fisher Scientific | NP0004 |
| NuPAGE™ 4-12% Bis-Tris Protein Gel | Thermo Fisher Scientific | NP0322BOX |
| SeeBlue™ Plus2 pre-stained protein standard | Thermo Fisher Scientific | LC5925 |
| Amersham™ Protran™ Premium 0.45µm NC membrane | GE Healthcare | 10600096 |
| blocking-grade blocker | BioRad | 1706404 |
| Pierce™ ECL Western Blotting Substrate | Thermo Fisher Scientific | 32109 |
| Restore™ PLUS Western Blot Stripping Buffer | Thermo Fisher Scientific | 46430 |
| Wizard® SV Gel and PCT Clean-Up System | Promega | A9281 |
| TOPO® TA Cloning® Kit for Sequencing | Thermo Fisher Scientific | 450030 |
| DH5α Competent *E. coli* | New England Biolabs | C2987 |
| Carbenicillin | Fisher | BP2658-5 |
| QIAprep® Spin Miniprep Kit | QIAGEN | 27104 |
| *Eco*RI | New England Biolabs | R0101 |
| *Bbs*I-HF | New England Biolabs | R3539S |
| One Shot™ Stbl3™ Chemically Competent *E. coli* | Thermo Fisher Scientific | C737303 |
| pSpCas9n-2A-Puro plasmid (PX462) | Addgene | 48141 |
