# Supplementary material for: Engineering synucleinopathy‐resistant human dopaminergic neurons by CRISPR‐mediated deletion of the SNCA gene
Source: Eur J Neurosci. 2018 Dec 21;49(4):510–24. doi: 10.1111/ejn.14286 (PMC6492083; doi:10.1111/ejn.14286)
Supplement: Supplementary file 2 [file EJN-49-510-s002.docx]

Engineering synucleinopathy-resistant human dopaminergic neurons by CRISPR-mediated deletion of the SNCA gene

Kunath, Tilo ; Chen, Yixi; Singh Dolt, Karamjit; Kriek, Marco; Baker, Terry; Downey, Patrick; Drummond, Nicola;

Canham, Maurice; Natalwala, Ammar; Rosser, Susan

_____________________________________________________________________________________

| Review timeline: | Submission date: 28/05/18  Editorial Decision: Major Revision  Revision Received: 02/10/18  Editorial Decision: Minor Revision  Revision Received: 12/11/18  Accepted: 16/11/18 |  |
| --- | --- | --- |
|  |  |  |

Editor: Paul Bolam

Reviewer 1: Natalie Connor-Robson

Reviewer 2: Eilis Dowd

_____________________________________________________________________________________

1st Editorial Decision	02/07/18

_____________________________________________________________________________________

Engineering synucleinopathy-resistant human dopaminergic neurons by CRISPR-mediated deletion of the SNCA gene 

Dear Dr. Kunath, 

Your manuscript has been reviewed by external reviewers as well as by the Editorial team.   

As you can see, both reviewers recognise the importance of your study and that it is a fitting contribution to the Special Issue in memory of Tom.  Both raise a few points however, that will need to be addressed in a revised version of the manuscript.  The comments of reviewer 2 will be relatively straight-forward to deal with requiring expansion of the Discussion.  Reviewer 1 raises more challenging issues including, the sparsity of statistical analyses, the failure to convincingly identify the cells as dopaminergic and the interpretation of some of the immuno.  Please carefully address each of the points that they have raised.   

In addition we noted the following points that need to be addressed in the revised version.   
- Please move the Methods section to correct position i.e. after the Introduction. 
- I suspect we will need higher resolution figures for publication. 
- Please provide a list of abbreviations and a data statement. 
- Please provide a Graphical Abstract. 
- For the statistical that you already have and any new data, please describe in the methods section and include in both results and figure legends.  Please ensure that you adhere to EJN guidelines, notably report precise P values.   
- The scale bars in the figures will need to be thicker. 

When revising the manuscript, please embolden or underline major changes to the text so they are easily identifiable and DO NOT leave 'track change' formatting marks in your paper.  Please ensure that you provide a text and a figure file for the Graphical Abstract (as detailed in the instructions below).  When carrying out your revisions please refer to the checklist below and visit the EJN author guidelines at www.ejneuroscience.org 

When finalized, please upload your complete revised manuscript onto the website, as a Word file (.doc, or .docx).  Please also ensure that a complete set of tables and figures is included as separate files, even if these have not changed from the originals.  At this stage it is necessary to provide high resolution figures.  Please see important instructions below. 

Please go into https://mc.manuscriptcentral.com/ejn - Author Centre - manuscripts with decisions where you will find a 'create a revision' link under 'actions'. We ask that you please indicate the way in which you have responded to the points raised by the Editors and Reviewers in a letter. Please upload this response letter as a separate Word (.doc or PDF) file using the file designation "Authors' Response to Reviewers" when uploading your manuscript files.  Please DO NOT submit your revised manuscript as a new one.  Also, please note that only the  Author who submitted the original version of the manuscript should submit a revised version. 

If you are able to respond fully to the points raised, we would be pleased to receive a revision of your paper within 12 weeks. 

Thank you for your support of this Special Issue of  EJN. 

Best wishes, 

Paul Bolam & John Foxe 
co-Editors in Chief, EJN 


Reviews: 

Reviewer: 1 

Comments to the Author 
The authors of this paper describe well-designed knockout strategy for the creation of a human ES cell line with the knockout of alpha-synuclein.  Due to the resistance of the line to alpha-synuclein pathology induced by addition of alpha-synuclein PFF to the cultures the authors suggest that this line might be useful for implantation therapy.  Previous work in this area in which patients received fetal mesencephalic transplants demonstrated alpha-synuclein pathology in the grafted cells.  This new ES line could therefore prove useful for such therapy in the future.  This reviewer recommends the paper should be accepted but with some major revisions. 

1. Throughout the paper, the authors fail to describe any statistical tests used or n numbers in their experiments.  Obviously, this is not acceptable for publication and therefore should be urgently addressed!  In particular figures 4 and 5 hhave legends that fail to describe the number of cells analysed or the number of fields of vision used for the analysis. 

2. In figure 1b and 2a it is unclear what the separate blots on the right hand side show and why they are separate from the blots on the left.  Please clarify this in the legend. 

3. Figure 2B claims to show mDA neurons with absolutely no evidence for it in the figure as the only markers used are generic neuronal markers. Although the authors suggest the newly created cell line would be differentiated to dopaminergic neurons for transplantation they fail to show in any of their figures that alpha-synuclein is indeed absent from TH positive neurons.  There are no immunos in the paper in which there is staining for both TH and alpha-synuclein. This reviewer feels this is an important point to address given the aims of the study and figure 2B should be amended to include TH staining. 

4. The characterisation of the ES cells that are differentiated to dopaminergic neurons is minimal.  The authors have only shown TH and FOXA2 immunoreactivity.  It would be nice to see other markers such as DAT and VMAT2 and to ensure that the knockout of alpha-synuclein does not affect the expression of such markers given their proposed use. 

5. In figure 2B the alpha-synuclein staining looks to be exclusively nuclear which is unexpected given it is primarily a synaptic protein.  Is staining evident in neurites at higher magnification?   

6. Figure 5b has quantification of pS129 in cultures treated with either PFFs or monomer however, there are no images of the monomer treated cells.  This should be amended to include them and also have the stats and n numbers clearly stated in the figure legend. 

7. In the discussion the authors state “the loss of alpha-synuclein in mice does not result in significant phenotypes…” but fail to reference any such papers.  The authors should amend this to include such references as Abeliovich 2000, Cabin et al and Anwar et al 2011. 

8. As a more general comment it would be nice to see some more function work e.g. dopamine HPLC, electrophys etc. on the knockout lines to see how the loss of alpha-synuclein might impact on the physiology of the cells especially if these were to be used for transplantation in the future.  The reviewer understands this is beyond the scope of this paper. 


Reviewer: 2 

Comments to the Author 
This manuscript by Yixi Chen, Tilo Kunath and colleagues is important in the field of cellular brain repair therapies for Parkinson’s because of the independent findings ~10 years ago by Patrik Brundin and Jeff Kordower that alpha-synuclein/Lewy body pathology from the diseased host brain can spread into transplanted fetal neurons. Indeed, these findings were the seminal findings that led to the prion hypothesis of Parkinson’s disease, a hypothesis that has gained considerable traction over the past decade. Because of host-to-graft disease spreading, there remained the possibility that cellular brain repair therapy for Parkinson’s would never meet its full potential. 

However, this manuscript describes 1) the use of CRISPR/Cas9 technology to generate human embryonic stem cells with deletion of one or both alleles of the alpha-synuclein gene, and 2) that these cells (once differentiated to a dopaminergic phenotype) are resistant to synucleinopathy induced by pre-formed alpha-synuclein fibrils (considered a model of cell-to-cell spread of alpha-synucleinopathy). This opens the possibility of using alpha-synucleinopathy resistant cells in reparative approaches for Parkinson’s thereby avoiding the issues of host-to-graft disease spreading. 

Overall, the work described is cleverly conceived and well designed, and the manuscript is very well written and clearly presented. There are just a few aspects of the manuscript which could be improved as follows: 

1. The discussion is very compact and would benefit from more discussion on: 

- Why the level of pS129-alpha-synuclein was similar in the SNCA +/- and SNCA -/- cells (Fig. 5). 
- With regard to the impact of alpha-synuclein deletion on hESC dopaminergic differentiation, it should be conceded more explicitly that the assays used here do not assess functionality of the neurons. More discussion on the role of alpha-synuclein in the normal development of dopaminergic neurons and the potential for affecting their function would also be useful here. 
- Can the authors explain how they might assess their cells’ resistance to alpha-synucleinopathy in an in vivo/preclinical setting? Are there animal models of host-to-graft disease spreading? 
- Are there any potential safety issues of CRISPR/Cas9 gene editing of cells prior to transplantation into patients’ brains? 
- Given the emphasis on the use of clinical grade hESCs as an “off-the-shelf” cell type for clinical transplantation, can the authors comment on how their gene editing may affect the translatability of the cells. 

2. There are a few typographical errors in the methods section: “which was consist” on Page 9; “Cells was” on Page 10, “was harvest” on Page 10,

____________________________________________________________________________________

Authors’ Response	02/10/18

_____________________________________________________________________________________

Reviewer: 1

The authors of this paper describe well-designed knockout strategy for the creation of a human ES cell line with the knockout of alpha-synuclein.  Due to the resistance of the line to alpha-synuclein pathology induced by addition of alpha-synuclein PFF to the cultures the authors suggest that this line might be useful for implantation therapy.  Previous work in this area in which patients received fetal mesencephalic transplants demonstrated alpha-synuclein pathology in the grafted cells.  This new ES line could therefore prove useful for such therapy in the future.  This reviewer recommends the paper should be accepted but with some major revisions.

1.	Throughout the paper, the authors fail to describe any statistical tests used or n numbers in their experiments.  Obviously, this is not acceptable for publication and therefore should be urgently addressed!  In particular figures 4 and 5 have legends that fail to describe the number of cells analysed or the number of fields of vision used for the analysis.

This is a completely valid point, and we apologize for this omission. We used the Mann-Whitney *U* test to check for statistical significance. We have also added all the n numbers, and total number of images analysed to Figure 6 legend (formerly Figure 5). All data for PFF seeding of *SNCA*^+/–^ and *SNCA*^–/–^ neurons in Figure 6 come from two independently CRISPR-targeted clones (Clones 4-4 and L5-4 for *SNCA*^+/–^, and Clones M1-4 and L5-3 for *SNCA*^–/–^). We have also performed the targeting of *SNCA* in a triplication SNCA iPSC clone to produce alpha-synuclein-null iPSCs and neurons, and the resistance to aSyn PFFs is also observed in the system. This data is presented in a new Figure 7.

2.	In figure 1b and 2a it is unclear what the separate blots on the right hand side show and why they are separate from the blots on the left.  Please clarify this in the legend.

The different DNA gels in Fig 1b and separate western blots in Fig 2a were due to the large number of clones analysed, which could not fit on a single gel. The different sizes of the blots made this rather unclear, and we apologize for this. We have now fixed this by making the blot sizes the same, and lined everything up. We have also moved the western blot data form Fig 2 to Fig 1 (Fig 1F), so it is in the same figure as the genotyping data. We have clarified all this in the new legend for Figure 1.

3.	Figure 2B claims to show mDA neurons with absolutely no evidence for it in the figure as the only markers used are generic neuronal markers. Although the authors suggest the newly created cell line would be differentiated to dopaminergic neurons for transplantation they fail to show in any of their figures that alpha-synuclein is indeed absent from TH positive neurons.  There are no immunos in the paper in which there is staining for both TH and alpha-synuclein. This reviewer feels this is an important point to address given the aims of the study and figure 2B should be amended to include TH staining.

We have now added co-immunostaining of TH and alpha-synuclein in new Figure 4 panels to specifically show that the *SNCA*-null TH neurons lack alpha-synuclein protein (Fig 4B). We have also added new RT-qPCR data in Figure 2 that demonstrate expression of mDA transcription factors NURR1 and SOX6 are significantly increased during differentiation into dopaminergic neurons. The highly pure CORIN FACS at day 16 (Fig 2B) confirms their ventral identify, and the vast majority of cells expressing FOXA2 (>95%) and TH at day 44 (Fig 3) confirms their midbrain dopaminergic identity. We have also transplanted our mDA cells of the parent hESC line, RC17, into 6-OHDA lesion rat model. Our mDA differentiation protocol produces dopaminergic neurons that innervate the rat striatum, and fully rescue the amphetamine-induced rotation defect caused by the lesion (figure below). We will now be testing whether the *SNCA*-null mDA cells can rescue the 6-OHDA lesion model with a similar efficacy as the wild-type neurons. This will be part of a subsequent manuscript.

4.	The characterisation of the ES cells that are differentiated to dopaminergic neurons is minimal.  The authors have only shown TH and FOXA2 immunoreactivity.  It would be nice to see other markers such as DAT and VMAT2 and to ensure that the knockout of alpha-synuclein does not affect the expression of such markers given their proposed use.

We have checked expression VMAT2 and DAT, as well as NURR1 and SOX6 in *SNCA*^+/–^ and *SNCA*^–/–^ neurons and do not observe any differences to wild-type neurons in new Figure 2 panels. This is in agreement with mouse data showing the *SNCA*^–/–^ animals do not have significant defects in formation of the nigro-striatal system or defects in any other dopaminergic neurons. This does not exclude more subtle defects in synaptic biology, which are being investigated at present. The parental hESCs, RC17, from which the knock-outs were made, show excellent dopaminergic differentiation potential and function when translplanted into the 6-OHDA lesion model (figure above).

5.	In figure 2B the alpha-synuclein staining looks to be exclusively nuclear which is unexpected given it is primarily a synaptic protein.  Is staining evident in neurites at higher magnification?

During development alpha-synuclein is nuclear and cytoplasmic, and only becomes localised to synapses at relatively late stages of neuronal differentiation. In the human, this occurs at 16 weeks gestation (Gavin et al., 2001). Our cultures are between 6 – 11 weeks (44 – 80 days) of differentiation, so we expect alpha-synuclein to be nuclear and cytoplasmic at these stages. We explicitly state this now, and reference Gavin et al.

6.	Figure 5b has quantification of pS129 in cultures treated with either PFFs or monomer however, there are no images of the monomer treated cells.  This should be amended to include them and also have the stats and n numbers clearly stated in the figure legend.

We have now added images of the monomer-treated and vehicle-treated cultures in new Supplementary Figure S6.

7.	In the discussion the authors state “the loss of alpha-synuclein in mice does not result in significant phenotypes…” but fail to reference any such papers.  The authors should amend this to include such references as Abeliovich 2000, Cabin et al and Anwar et al 2011.

This is a very valid point, and apologies for not writing a more thorough Discussion. This has now been rectified by referencing and discussing Abeliovich 2000, Cabin et al and Anwar et al 2011, in addition to Al-Wandi et al 2010 which demonstrated reduced DA content in *SNCA*^–/–^ mouse striatum. We also discuss and reference literature showing *SNCA*^–/–^ mice have increased resistance to MPTP toxin (Dauer et al., 2002; Robertson et al., 2004), and data showing *SNCA*^–/–^ mice are highly susceptible to West Nile virus (Beatman et al., 2015).

8.	As a more general comment it would be nice to see some more function work e.g. dopamine HPLC, electrophys etc. on the knockout lines to see how the loss of alpha-synuclein might impact on the physiology of the cells especially if these were to be used for transplantation in the future.  The reviewer understands this is beyond the scope of this paper.

We completely agree with the reviewer that functional work on dopamine metabolism, synaptic biology, and other phenotyping of *SNCA*^–/–^ neurons, such as rescue of the 6-OHDA lesion model, and susceptibility to neuroinvasive viruses, are needed. Some of these experiments are being conducted at present, and this data will be part of a subsequent publication.


Reviewer: 2

This manuscript by Yixi Chen, Tilo Kunath and colleagues is important in the field of cellular brain repair therapies for Parkinson’s because of the independent findings ~10 years ago by Patrik Brundin and Jeff Kordower that alpha-synuclein/Lewy body pathology from the diseased host brain can spread into transplanted fetal neurons. Indeed, these findings were the seminal findings that led to the prion hypothesis of Parkinson’s disease, a hypothesis that has gained considerable traction over the past decade. Because of host-to-graft disease spreading, there remained the possibility that cellular brain repair therapy for Parkinson’s would never meet its full potential. 

However, this manuscript describes 1) the use of CRISPR/Cas9 technology to generate human embryonic stem cells with deletion of one or both alleles of the alpha-synuclein gene, and 2) that these cells (once differentiated to a dopaminergic phenotype) are resistant to synucleinopathy induced by pre-formed alpha-synuclein fibrils (considered a model of cell-to-cell spread of alpha-synucleinopathy). This opens the possibility of using alpha-synucleinopathy resistant cells in reparative approaches for Parkinson’s thereby avoiding the issues of host-to-graft disease spreading.

Overall, the work described is cleverly conceived and well designed, and the manuscript is very well written and clearly presented. There are just a few aspects of the manuscript which could be improved as follows:

1.	The discussion is very compact and would benefit from more discussion on:
-	Why the level of pS129-alpha-synuclein was similar in the SNCA +/- and SNCA -/- cells (Fig. 5).

We think this has to do with the length of time of the assay, 5 weeks, a longer assay may reveal more differences between the *SNCA*^+/–^ and *SNCA*^–/–^ neurons. We have added more data and experiments to this data set, and now there is a small, but significant, difference between the *SNCA*^+/–^ and *SNCA*^–/–^ neurons. This data is in a new Figure 6.

-	With regard to the impact of alpha-synuclein deletion on hESC dopaminergic differentiation, it should be conceded more explicitly that the assays used here do not assess functionality of the neurons. More discussion on the role of alpha-synuclein in the normal development of dopaminergic neurons and the potential for affecting their function would also be useful here.
-	Can the authors explain how they might assess their cells’ resistance to alpha-synucleinopathy in an in vivo/preclinical setting? Are there animal models of host-to-graft disease spreading?
-	Are there any potential safety issues of CRISPR/Cas9 gene editing of cells prior to transplantation into patients’ brains?
-	Given the emphasis on the use of clinical grade hESCs as an “off-the-shelf” cell type for clinical transplantation, can the authors comment on how their gene editing may affect the translatability of the cells.

We have significantly increased the length and depth of the Discussion by referring to a number of studies investigating the phenotype of *SNCA*^–/–^ mice and discussed the implications of the work on the *SNCA*^–/–^ neurons we have created. We also extended discussion of how we plan to functionally test the *SNCA*^–/–^ neurons in synaptic and in vivo assays. We also added a discussion of how the *SNCA*^–/–^ neurons could be tested in an in vivo model of host-to-graft spread and reference two models by Kordower et al 2011 and Hansen et al 2011. We have also referenced reviews discussing the application of CRISPR/Cas9 to medicine in general, and point out that gene-edited pluripotent stem cells are not in clinical trials yet for any indication, but will follow on from the diverse efforts to get CRISPR-manipulated cells, including T cells, to the clinic. The safety of any cell product will need to be tested empirically with animal models of tumourigenesis. We now highlight that the method we used (Cas9 nickase) and other emerging Cas9 methods have dramatically increased the precision and fidelity of the genome editing, and the risk of off-target deletions or insertions are now very low. We have also added new off-target analysis data in new Supplementary Figure S4 demonstrating the most likely off-target genomic sites are untouched by our Cas9n method.

2.	There are a few typographical errors in the methods section: “which was consist” on Page 9; “Cells was” on Page 10, “was harvest” on Page 10,

Thank you for this. This has been corrected now.

2^nd^ Editorial Decision	26/10.18

_____________________________________________________________________________________

Engineering synucleinopathy-resistant human dopaminergic neurons by CRISPR-mediated deletion of the SNCA gene 

Dear Dr. Kunath, 

Your revised manuscript was re-evaluated by the external reviewers as well as by the Editorial team and we are pleased to inform you that it is provisionally accepted for publication in the Special Issue of EJN after you have dealt with a few minor points (see below).  Once done we will send it off to production.   

- Please address the minor points raised by the reviewer concerning the statistical analysis 
- Please include a list of abbreviations 
- Any chance of converting the bar charts in fig. 2 to scatter plots? 
- Please include a Graphic abstract and legend. 

When finalised, please upload your complete revised manuscript onto the website as a Word (.doc or .docx), or .rtf file.  Please also ensure that a complete set of tables and figures is included as separate files, even if these have not changed from the originals.  At this stage it is necessary to provide high resolution figures.  Please see important instructions below. 

Please go into https://mc.manuscriptcentral.com/ejn - Author Centre - manuscripts with decisions where you will find a 'create a revision' link under 'actions'. We ask that you please indicate the way in which you have responded to the points raised by the Editors and Reviewers in a letter. Please upload this response letter as a separate Word (.doc) file using the file designation "Authors' Response to Reviewers" when uploading your manuscript files. Please DO NOT submit your revised manuscript as a new one.  Also, please note that only the  Author who submitted the original version of the manuscript should submit a revised version. 

If you are able to respond fully to the points raised, we shall be pleased to receive a revision of your paper within 30 days. 

Thank you for your support of this Special Issue of EJN. 

Best wishes, 

Paul Bolam & John Foxe 
co-Editors in Chief, EJN 

Reviews: 

Reviewer: 1 

Comments to the Author 
The Authors have answered the criticism and concerns of the original review satisfactorily. The authors have also added new additional data to the paper that is an excellent edition.  I therefore advise the paper should be accepted with minor revisions.   

This reveiwer asked that statistical testing was added to the paper in the methods and figure legends in the paper.  Although the authors respond in their comments and describe this it still isn't described in the materials and methods.  Could this please be added? 

The additional data added to figure 2 (2C) is a great addition however there are no stats described here.  The authors state "a significant  upregulation of dopaminergic markers....suggesting deletion of SNCA does not significantly affect the differentiation into mDA neurons." No stats are stated in the figure legend and although there is an upregulation compared to ES cells presumably the authors tested the significance between groups to see the affect of alpha-synuclein knockout on expression of key dopaminergic transcripts?  This should be made more clear and if there is a difference to WT then this should be commented on in the text as it is potentially very interesting.  For instance clone L5-3 looks to have reduced DAT and VMAT2 compared to WT. 

Reviewer: 2 

Comments to the Author 
All of my queries have been addressed.

_____________________________________________________________________________________

Authors’ Response	12/11/18

Dear Prof Bolam, 

Thank you for the conditional offer of acceptance, your instructions, and Reviewer #1 comments regarding our manuscript entitled “Engineering synucleinopathy-resistant human dopaminergic neurons by CRISPR-mediated deletion of the SNCA gene”. Below are our actions and responses: 
1. A list of abbreviation and graphical abstract with legend is now included. 
2. We have added a statement of statistical analysis to the Materials & Methods. 
3. We have clarified what the RT-qPCR data in showing in Fig 2. They are showing no major difference in dopaminergic differentiation of SNCA-null cells, in agreement with marker analysis in Fig. 3. The data is technical replicates, so we cannot perform statistics on this data. The word ‘significant’ was removed from all sentences referring to this data, and what the data represents has now been clarified in the Materials & Methods and legend of Figure 2. 
4. We have re-formatted Fig 7C data to a box and scatter plot to match data presented in Fig 6. 
5. We have corrected a few minor edits through-out the manuscripts 
6. We have made all raw data accessible via LabArchives DOI: 10.25833/8bj6-e156 

We hope this addresses all comments. 

Yours faithfully, 
Tilo Kunath
